# A Bio-Inspired Vibration Energy Harvesting System with Internal Resonance and Slapping Mechanism for Enhanced Low-Frequency Power Generation

**DOI:** 10.3390/s25237222

**Published:** 2025-11-26

**Authors:** Yi-Ren Wang, Shian-Hsuan Chen, Su-Sheng Ma

**Affiliations:** Department of Aerospace Engineering, Tamkang University, Tamsui District, New Taipei City 25137, Taiwan160334@mail.tku.edu.tw (S.-S.M.)

**Keywords:** bio-inspired energy harvesting, slapping mechanism, piezoelectric vibration energy harvester, internal resonance

## Abstract

This study presents the development and validation of a bio-inspired vibration energy harvesting system, termed the Bio-Inspired Epiphytic-Plant Slapping Vibration Energy Harvesting System (BIS-VEHS). Inspired by the swaying and slapping behavior of epiphytic plants, the system integrates a circular plate, an elastic beam, a surface-bonded piezoelectric patch (PZT), and a lever-type slapping mechanism to enhance energy conversion. A nonlinear beam model is established and analyzed using the method of multiple scales, through which a 1:3 internal resonance between the first and third bending modes is identified as a key mechanism for promoting energy transfer from higher to lower modes. Time responses are obtained via numerical simulation using the Runge–Kutta method, and the model is validated experimentally. The results confirm that both internal resonance and the slapping mechanism significantly increase the harvested voltage compared with non-resonant and non-slapping configurations. Comparative tests under different excitation modes and plate configurations show good agreement between theory and experiment, with most discrepancies within 10%. These findings demonstrate that the BIS-VEHS is a promising candidate for sustainable low-frequency vibration energy harvesting, particularly for autonomous low-power sensor applications.

## 1. Introduction

Vibrations are ubiquitous in nature and engineering systems, and if effectively harnessed, they offer a sustainable means of addressing energy scarcity. Consequently, vibration energy harvesting (VEH) has emerged as a promising technology for powering low-energy devices. Compared with traditional batteries, which require periodic replacement and pose environmental concerns, VEH systems are self-sustaining, environmentally friendly, and suitable for long-term autonomous operation. Inspiration for the present study comes from the motion of epiphytic plants, whose flexible stems sway in the wind while their leaves and branches occasionally strike surrounding structures. Mimicking this natural phenomenon, a piezoelectric-based energy harvester can be designed by integrating a circular plate and an elastic beam to emulate leaf–stem motion, with a lever-type mechanism to introduce periodic slapping on the piezoelectric patch. This configuration enables simultaneous harvesting from both structural deformation and impact forces, resulting in higher energy conversion efficiency. The proposed system is termed the Bio-Inspired Epiphytic-Plant Slapping Vibration Energy Harvesting System (BIS-VEHS).

Recent efforts in bio-inspired design have demonstrated how natural motion can guide the development of efficient vibration-based systems. Bio-inspired energy harvesters have drawn from mechanisms such as insect or bird wing flapping and plant oscillations in the wind, offering novel strategies for vibration-to-electricity conversion. Teng et al. [1] reviewed various renewable energy sources in nature and showed that vibration-based piezoelectric harvesters achieve maximum output when excitation frequencies align with resonance conditions. Similarly, Yan et al. [2] categorized bio-inspired vibration isolators into stiffness-tuning, auxiliary mass, and damping types, highlighting their potential for low-energy and resilient control systems. Huang et al. [3] developed a dual-stage vibration isolation model (BI-DSVI) and optimized its dynamic parameters for enhanced performance, while Zhou et al. [4] proposed a Bilateral Supported Bio-Inspired Anti-Vibration (BBAV) structure with improved low-frequency isolation. These studies collectively emphasize the potential of bio-inspired motion as a pathway toward advanced energy harvesting and vibration-control technologies.

In addition to bio-inspired motion-based designs, the direct use of living plants as power sources has recently attracted considerable attention. Choo and Dayou [5] first demonstrated that electricity can be harvested from plants by embedding metal electrodes to exploit ionic flow within plant tissues. Their experiments showed that a copper–zinc electrode pair inserted into Aloe vera generated up to 0.95 V, confirming the feasibility of biological power generation. Ying and Dayou [6] further analyzed this process, attributing the generated current to electrochemical reactions between dissimilar electrodes and plant electrolytes, and proposed a corresponding theoretical model. Pechsiri and Puengsungwan [7] extended this concept to plant–microbial fuel cells (PMFCs) for IoT and wireless sensor applications, harvesting 0.37–0.65 V from avocado roots using Cu–Al electrodes. Greenman et al. [8] comprehensively reviewed PMFC technologies, emphasizing their renewability, zero-pollution operation, and potential for continuous biocharging of low-power devices.

Other plant-inspired designs focus on structural imitation rather than living energy conversion. Wang et al. [9] showed that incorporating vein-like geometries into piezoelectric sheets significantly enhances power output under wind excitation, while Qian et al. [10] developed a bistable harvester inspired by the snap-through motion of the Venus flytrap, achieving low-cost and high-efficiency energy collection. These studies highlight the wide spectrum of plant-based inspiration—from biological electrochemistry to mechanical analogs—for sustainable energy harvesting.

To accurately model the elongated, stem-like elastic beams of the BIS-VEHS, it is essential to consider their nonlinear vibration characteristics, as large deflections occur under low-frequency excitation. Lenci et al. [11] investigated the nonlinear vibrations of a double-layer beam with a nonlinear elastic interface, showing that boundary conditions and stiffness ratios strongly affect natural frequencies. Kharazan et al. [12] analyzed cantilever beams with breathing cracks, deriving a polynomial approximation of bilinear stiffness using the Stone–Weierstrass theorem. Chouvion [13] applied a wave-propagation framework to structures with localized nonlinearities, demonstrating boundary-condition-dependent modal behavior. Beyond geometric nonlinearity, internal resonance plays a crucial role in amplifying low-frequency response. Nayfeh and Mook [14] described internal resonance as the condition where modal frequencies are integer multiples, allowing high-frequency excitation to transfer energy to lower modes. Building on this principle, Thai et al. [15] developed a flexoelectric microbeam model exhibiting nonlinear resonance behavior influenced by geometry and resistance, while Sahoo [16] analyzed 3:1 internal resonance in hinged–clamped beams, showing strong energy transfer between modes. Wang and Chen [17] further demonstrated similar modal coupling in fluid-conveying pipelines using nonlinear beam theory and Hamilton’s principle. These foundational studies provide the theoretical basis for identifying and exploiting internal resonance in the BIS-VEHS to enhance its energy-harvesting efficiency.

In recent years, a variety of advanced vibration energy harvesters and self-powered sensor systems have been developed to broaden the functional scope of traditional piezoelectric designs. The following studies represent recent progress in nonlinear, bio-integrated, and hybrid energy-harvesting technologies, providing the context for the present BIS-VEHS development. Recent studies on variable damping mechanisms under interval-parameter uncertainties (Sofi et al. [18]) further highlight the importance of precise modeling of damping effects in energy harvesting systems. Chen et al. [19] propose a novel nonlinear electromagnetic vibration energy harvester designed for ultra-low-frequency and small-amplitude environments, incorporating dual helical-plane springs to introduce nonlinear stiffness and multiple Halbach magnet arrays to enhance the magnetic flux. Their experimental prototype achieved a peak output power of ~14 mW at only 0.5 g excitation and exhibited a resonance bandwidth of about 3 Hz. This study demonstrates that by combining nonlinear mechanical elements with compact electromagnetic transduction, broadband and high-efficiency harvesting can be achieved in real-world low-vibration scenarios—an approach that maps directly onto the structural nonlinearity and internal resonance strategies explored in the present BIS-VEHS work. Clementi et al. [20] present a novel bio-generator system in which the membrane potential of a single living muscle fiber is harvested via an embedded RLC circuit, achieving a maximum output voltage of around −70 mV and enabling wireless remote temperature sensing. This work significantly broadens the domain of self-powered sensors by exploiting cellular electro-chemical potentials rather than mechanical vibrations or ambient flows, thereby providing a useful parallel to the mechanical harvester in the present BIS-VEHS study. Guo et al. [21] developed a hydrogel-based self-powered sensor system designed for outdoor plant monitoring: the hydrogel harvests energy and produces a stable DC output with an average power density of 1.9 W m^−3^ over 60 days, enabling leaf-water-content sensing in situ. This work contributes to the ecosystem of plant-integrated energy harvesters and further supports the motivation for integrating bio-inspired mechanical systems like the BIS-VEHS for self-sustained sensing in natural or agricultural settings. Qu et al. [22] offer a comprehensive review of recent advances in vibrational energy harvesters, covering electromagnetic, piezoelectric, electrostatic, and magnetostrictive transduction mechanisms, and discuss future trends such as hybridization, broadband response, and application in self-powered sensing networks. Their analysis highlights the rise in design strategies that exploit structural nonlinearity and modal coupling to expand bandwidth and improve output, which directly supports the design rationale of the present BIS-VEHS system based on internal resonance coupling and slapping impact. Incorporating this review strengthens the state-of-the-art section by linking the current work to broader trends in VEH design for sensors. 

The slapping mechanism has also been shown to significantly enhance piezoelectric energy conversion. Wang and Chu [23] utilized airflow beneath rotorcraft blades to drive a small wind turbine that produced a rotating magnetic field, which in turn induced periodic repulsive forces on magnets attached to elastic steel plates. These plates repeatedly struck the piezoelectric layer, increasing both vibration amplitude and power output. Their results demonstrated that dual slapping plates yielded substantially higher energy conversion efficiency than single-plate configurations. Building on this concept, Wang et al. [24,25] proposed a double elastic steel (DES) VEH consisting of two parallel cantilever beams. By analyzing the transverse vibration and slapping behavior of fixed–fixed beams, they identified optimal piezoelectric film locations corresponding to peak deformation nodes. These studies collectively underscore the critical role of impact-induced slapping in amplifying strain energy and enhancing piezoelectric conversion efficiency.

**Figure 1:**
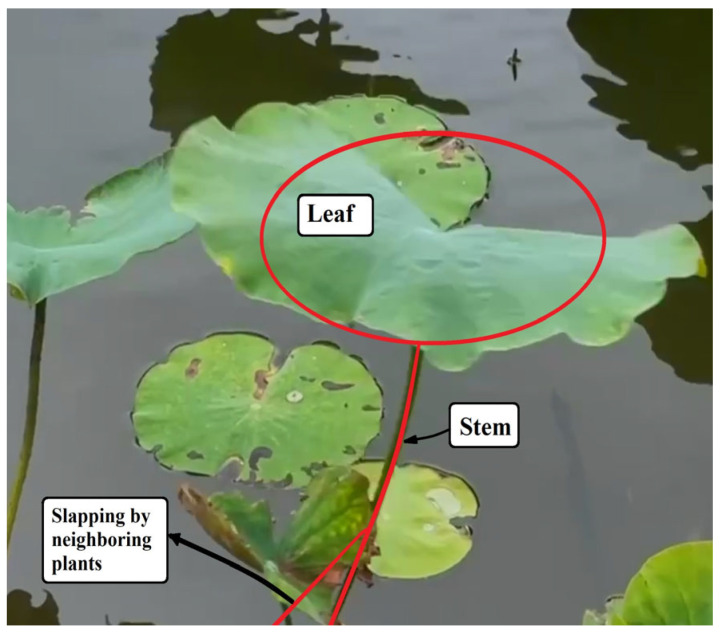
An epiphytic plant observed on campus, showing a circular leaf slender stalk.

**Figure 2:**
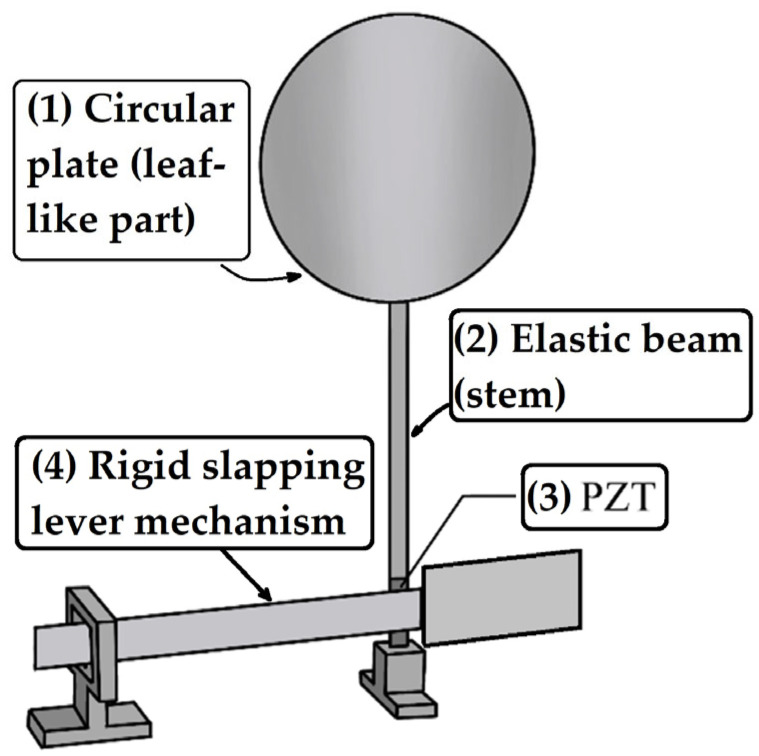
Bio-inspired BIS-VEHS structure attached to a corresponding to the plant’s morphology. Tags: (1) Circular plate (leaf-like part), (2) Elastic beam (stem), (3) Piezoelectric patch (PZT) at the root, and (4) Rigid slapping lever mechanism.

Careful observation of natural plant ecosystems (Figure 1) reveals that many species feature slender, flexible stems with circular leaf-like structures surrounded by parasitic or attached branches. When exposed to wind, these plants exhibit harmonic swaying motions, and their bases are intermittently struck by nearby epiphytic branches or vines. This dynamic interaction inspired the design of a bio-inspired vibration energy harvesting system. This proposed configuration consists of a circular plate representing the leaf, attached to an elongated, flexible stalk-like beam with a piezoelectric patch (PZT) bonded at its base (Figure 1). Adjacent to the main structure, a rigid lever mechanism equipped with a rectangular panel is installed to generate periodic slapping impacts on the PZT (Figure 2). The combined design—termed the Bio-Inspired Epiphytic-Plant Slapping Vibration Energy Harvesting System (BIS-VEHS)—converts ambient vibration into electrical energy through two concurrent processes: structural oscillation of the leaf–stalk assembly and impact-induced slapping on the piezoelectric layer.

The BIS-VEHS introduces two major innovations. First, the integration of a synchronized lever-type slapping mechanism enables hybrid energy conversion through vibration and impact. Second, the optimized geometry of the flexible beam and circular plate—specifically their mass, length, and moment of inertia—is tuned to satisfy internal resonance conditions, allowing efficient energy transfer between vibration modes. In nonlinear systems exhibiting internal resonance, energy flows from high-frequency to low-frequency modes; thus, the BIS-VEHS ensures that low-frequency vibrations consistently dominate regardless of excitation source. This characteristic maximizes energy harvesting efficiency by concentrating power generation within the low-frequency band most relevant to ambient vibrations. Consequently, the BIS-VEHS offers a compact, adaptive, and high-performance solution for harvesting low-frequency vibration energy.

## 2. Establishment of Theoretical Model

### 2.1. Derivation of Nonlinear Equations of Motion

**Figure 3:**
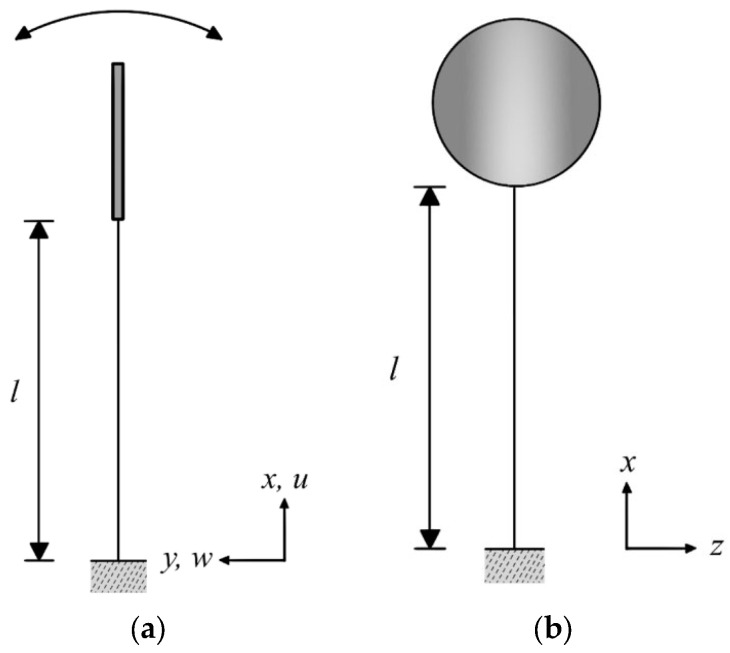
Coordinate definition of the fixed–free beam system: (**a**) side view and (**b**) front view, where l  denotes the beam length and the arrows indicate the coordinate axes.

The nonlinear transverse beam equation of motion is first derived using Hamilton’s principle. The definition of the relevant coordinates is shown in the side view in Figure 3a,b, which also present the front view of this fixed–free beam system.

The kinetic energy (*T*) of this system is expressed as follows:

(1) $$T=\int_{0}^{l}\left[\frac{1}{2}m_{b}{\left(\dot{u}+y\frac{\partial^{2}w}{\partial t\partial x}\right)}^{2}+\frac{1}{2}m_{b}{\dot{w}}^{2}\right]dx$$

where *m_b_* is the beam mass. The potential energy (*U*) of this system is expressed as follows:

(2) $$U=\int_{0}^{l}\frac{1}{2}EA_{b}\eta^{2}dx-\int_{0}^{l}qwdx$$

where η represents the Von Kármán nonlinear strain and is expressed as $\eta =\frac{\partial u}{\partial x}+\frac{1}{2}{\left(\frac{\partial w}{\partial x}\right)}^{2}+y\left(\frac{\partial^{2}w}{\partial x^{2}}\right)$, *A_b_* is the beam cross-sectional area, and *q* is the distributed external force. Using Hamilton’s principle, the equations of motion for this nonlinear system in the *u*-direction and *w*-direction can be derived as follows:

*u*-direction:

(3) $$m_{b}\ddot{u}-EA_{b}\left(u^{\prime \prime }+w^{\prime \prime }w^{\prime }\right)=0$$

*w*-direction:

(4) $$m_{b}\ddot{w}-m_{b}y^{2}{\ddot{w}}^{\prime \prime }+EA_{b}y^{2}w^{iv}-EA_{b}\left(u^{\prime \prime }w^{\prime }+u^{\prime }w^{\prime \prime }+\frac{3}{2}w^{\prime \prime }{w^{\prime }}^{2}\right)-q=0$$

where ${\left(\;\right)}^{\prime }$ represents $\frac{d}{dx}$, $\overset{\cdot }{\left(\;\right)}$ denotes $\frac{d}{dt}$.

The dimensionless forms of the equations in the *u*- and *w*- directions are as follows:

(5) $${\bar{u}}^{**}-\left(\frac{\partial^{2}\bar{u}}{\partial {\bar{x}}^{2}}+\frac{\partial \bar{w}}{\partial \bar{x}}\frac{\partial^{2}\bar{w}}{\partial {\bar{x}}^{2}}\right)+\mu_{u}u^{*}=0$$

(6) $${\bar{w}}^{**}-{\bar{I}}_{b}\frac{\partial^{2}{\bar{w}}^{**}}{\partial {\bar{x}}^{2}}+\frac{\partial^{4}\bar{w}}{\partial {\bar{x}}^{4}}-\left({\bar{u}}^{\prime \prime }{\bar{w}}^{\prime }+{\bar{u}}^{\prime }{\bar{w}}^{\prime \prime }+\frac{3}{2}{\bar{w}}^{\prime \prime }{{\bar{w}}^{\prime }}^{2}\right)-\bar{q}+\mu_{w}{\bar{w}}^{*}=0$$

where $\bar{u}=\frac{u}{l}$, $\bar{w}=\frac{w}{l}$, ${\bar{I}}_{b}=\frac{m_{b}y^{2}}{m_{b}l^{2}}$, ${\left(\;\right)}^{\prime }$ represents $\frac{d}{d\bar{x}}$, ${\left(\;\right)}^{*}$ denotes $\frac{d}{d\tau }$, $\bar{q}=\frac{q}{m_{b}l\omega^{2}}$ represents the distributed load, and $\omega =\sqrt{\frac{EI}{m_{b}l^{4}}}$. 

The dimensionless boundary conditions for $\bar{u}$ are written as follows:

(7) $$\bar{u}\left(0,\tau \right)=0,\;{\bar{u}}^{\prime }\left(1,\tau \right)=0$$

Substituting Equation (7) into Equation (5) and integrating yields the following result:

(8) $$\bar{u}=-\frac{1}{2}\int_{0}^{\bar{x}}{{\bar{w}}^{\prime }}^{2}d\bar{x}+C_{1}\left(\bar{x}\right)+C_{2}\left(\bar{x}\right)$$

Here, *C*_1_(*x*) and *C*_2_(*x*) can be determined from the boundary conditions. Substituting the above expressions into Equation (6) gives the equations of motion for the beam system:

(9) $${\bar{w}}^{**}-{\bar{I}}_{b}\frac{\partial^{2}{\bar{w}}^{**}}{\partial {\bar{x}}^{2}}+\frac{\partial^{4}\bar{w}}{\partial {\bar{x}}^{4}}+\frac{1}{2}({\bar{w}}^{\prime }\int_{1}^{\bar{x}}(\int_{0}^{\bar{x}}{{\bar{w}}^{\prime }}^{2}d\bar{x}{)}^{**}d\bar{x}{)}^{\prime }-\bar{q}+\mu_{w}{\bar{w}}^{*}=0$$

### 2.2. Natural Frequency

The method of multiple scales (MOMS) proposed by Nayfeh and Mook [14] is employed to analyze the nonlinear equations of motion, where the time scale is separated into fast-varying and slow-varying components. Let $T_{0}=\tau$ represent the fast-varying component, and $T_{1}=\varepsilon^{2}\tau$ the slow-varying component, assuming $\bar{w}=\varepsilon^{1}{\bar{w}}_{0}+\varepsilon^{3}{\bar{w}}_{1}$, where ε denotes the time scale of small perturbations. Differentiating with respect to time τ, yields the following:

(10) $$\frac{\partial \bar{w}}{\partial \tau }=\varepsilon^{1}\frac{\partial {\bar{w}}_{0}}{\partial T_{0}}+\varepsilon^{3}\frac{\partial {\bar{w}}_{0}}{\partial T_{1}}+\varepsilon^{3}\frac{\partial {\bar{w}}_{1}}{\partial T_{0}}+\varepsilon^{5}\frac{\partial {\bar{w}}_{1}}{\partial T_{1}}+\ldots ,\;\;\frac{\partial^{2}\bar{w}}{\partial \tau^{2}}=\varepsilon^{1}\frac{\partial^{2}{\bar{w}}_{0}}{\partial T_{0}^{2}}+\varepsilon^{3}\frac{\partial^{2}{\bar{w}}_{1}}{\partial T_{1}^{2}}+2\varepsilon^{3}\frac{\partial^{2}{\bar{w}}_{0}}{\partial T_{0}\partial T_{1}}+2\varepsilon^{5}\frac{\partial^{2}{\bar{w}}_{1}}{\partial T_{0}\partial T_{1}}+\ldots$$

By setting the damping term as $\varepsilon^{2}\mu_{w}$, Equation (9) can be expressed as a multiple-time-scale equation:

(11) $$\begin{array}{l} \left(\varepsilon^{1}\frac{\partial^{2}{\bar{w}}_{0}}{\partial T_{0}^{2}}+\varepsilon^{3}\frac{\partial^{2}{\bar{w}}_{1}}{\partial T_{1}^{2}}+2\varepsilon^{3}\frac{\partial^{2}{\bar{w}}_{0}}{\partial T_{0}\partial T_{1}}+2\varepsilon^{5}\frac{\partial^{2}{\bar{w}}_{1}}{\partial T_{0}\partial T_{1}}+\ldots \right)\\ \;-{\bar{I}}_{b}\frac{\partial^{2}}{\partial {\bar{x}}^{2}}\left(\varepsilon^{1}\frac{\partial^{2}{\bar{w}}_{0}}{\partial T_{0}^{2}}+\varepsilon^{3}\frac{\partial^{2}{\bar{w}}_{1}}{\partial T_{1}^{2}}+2\varepsilon^{3}\frac{\partial^{2}{\bar{w}}_{0}}{\partial T_{0}\partial T_{1}}+2\varepsilon^{5}\frac{\partial^{2}{\bar{w}}_{1}}{\partial T_{0}\partial T_{1}}+\ldots \right)\\ \;+\left(\varepsilon {\bar{w}}_{0}^{iv}+\varepsilon^{3}{\bar{w}}_{1}^{iv}\right)+\frac{1}{2}\varepsilon^{3}({{\bar{w}}^{\prime }}_{0}\int_{1}^{\bar{x}}(\int_{0}^{\bar{x}}{\bar{w}}_{0}^{\prime 2}d\bar{x}{)}^{**}d\bar{x}{)}^{\prime }\;+\varepsilon^{2}\mu_{w}\left(\varepsilon \frac{\partial {\bar{w}}_{0}}{\partial T_{0}}+\varepsilon^{3}\frac{\partial {\bar{w}}_{1}}{\partial T_{0}}+\varepsilon^{3}\frac{\partial {\bar{w}}_{0}}{\partial T_{1}}+\ldots \right)-\bar{q}=0 \end{array}$$

Since ε is a very small value, we neglect the effects of higher-order terms $\varepsilon^{5},\varepsilon^{7}$… Next, we decompose Equation (11) into components of $\varepsilon^{1}$ and $\varepsilon^{3}$ terms to facilitate the subsequent analysis.

$\varepsilon^{1}$ terms:

(12) $$\frac{\partial^{2}{\bar{w}}_{0}}{\partial T_{0}^{2}}-{\bar{I}}_{b}\frac{\partial^{2}}{\partial {\bar{x}}^{2}}\left(\frac{\partial^{2}{\bar{w}}_{0}}{\partial T_{0}^{2}}\right)+\left({\bar{w}}_{0}^{iv}\right)=0$$

$\varepsilon^{3}$ terms:

(13) $$\frac{\partial^{2}{\bar{w}}_{1}}{\partial T_{1}^{2}}+2\frac{\partial^{2}{\bar{w}}_{0}}{\partial T_{0}\partial T_{1}}-{\bar{I}}_{b}\frac{\partial^{2}}{\partial {\bar{x}}^{2}}\left(\frac{\partial^{2}{\bar{w}}_{1}}{\partial T_{1}^{2}}+2\frac{\partial^{2}{\bar{w}}_{0}}{\partial T_{0}\partial T_{1}}\right)+\left({\bar{w}}_{1}^{iv}\right)+\frac{1}{2}({{\bar{w}}^{\prime }}_{0}\int_{1}^{\bar{x}}(\int_{0}^{\bar{x}}(\frac{\partial^{3}{\bar{w}}_{0}}{\partial T_{0}^{2}\partial \bar{x}}{)}^{2}d\bar{x})d\bar{x}{)}^{\prime }+\mu_{w}\left(\frac{\partial {\bar{w}}_{0}}{\partial T_{0}}\right)-\bar{q}=0$$

**Figure 4:**
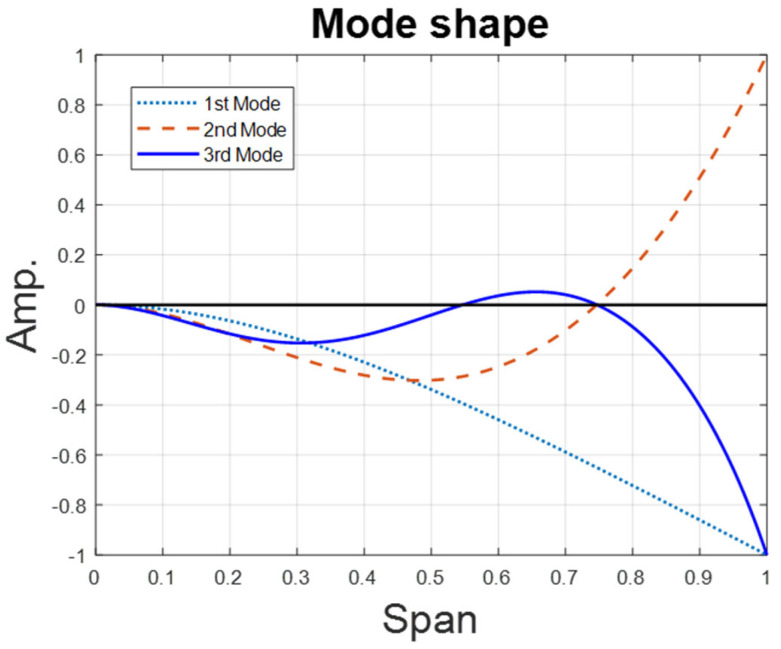
The first three mode shapes of the fixed–free beam.

By applying the separation of variables and the fixed–free boundary conditions, the vibration mode shapes of the system can be obtained as follows (see Appendix A):

(14) $$\begin{array}{l} \phi_{n}=\left[\cos(\alpha_{n}\bar{x})-\cosh(\alpha_{n}\bar{x})\right]\\ \;+\frac{-\cos\alpha_{n}-\cosh\alpha_{n}+{\bar{I}}_{A}\alpha_{n}^{3}\sin\alpha_{n}+{\bar{I}}_{A}\alpha_{n}^{3}\sinh\alpha_{n}}{\sin\alpha_{n}+\sinh\alpha_{n}+{\bar{I}}_{A}\alpha_{n}^{3}\cos\alpha_{n}-{\bar{I}}_{A}\alpha_{n}^{3}\cosh\alpha_{n}}\left[\sin\alpha_{n}\bar{x}\right.\;\left.-\sinh\alpha_{n}\bar{x}\right] \end{array}$$

where ${\bar{I}}_{A}$ is the dimensionless mass moment of inertia of the circular plate (leaf). Figure 4 shows the first three vibration modes of this system. 

Further, the displacements of each time scale are assumed to be

(15) $${\bar{w}}_{0}=\sum_{n=1}^{\infty }\phi_{n}\xi_{0n},\;{\bar{w}}_{1}=\sum_{n=1}^{\infty }\phi_{n}\xi_{1n}$$

Substituting Equation (15) into Equations (12) and (13) gives

(16) $$\sum_{n=1}^{\infty }\left(\phi_{n}-{\bar{I}}_{b}\phi_{n}^{\prime \prime }\right)\xi_{0n}^{**}+\sum_{n=1}^{\infty }\phi_{n}^{iv}\xi_{0n}=0\;\;\sum_{n=1}^{\infty }\phi_{n}\xi_{1n}^{**}+2\frac{\partial }{\partial T_{1}}\sum_{n=1}^{\infty }\phi_{n}\xi_{0n}^{*}-\sum_{n=1}^{\infty }{\bar{I}}_{b}\phi_{n}^{\prime \prime }\xi_{1n}^{**}-2\frac{\partial }{\partial T_{1}}\sum_{n=1}^{\infty }{\bar{I}}_{b}\phi_{n}^{\prime \prime }\xi_{0n}+\sum_{n=1}^{\infty }\phi_{n}^{iv}\xi_{1n}$$

(17) $$-\frac{1}{2}\sum_{n=1}^{\infty }\phi_{n}^{\prime \prime }\phi_{n}^{\prime \prime }\phi_{j}^{\prime }\phi_{k}^{\prime }\xi_{0n}\xi_{0j}\xi_{0k}+\sum_{n=1}^{\infty }\mu_{w}\phi_{n}\xi_{0n}^{*}-{\bar{q}}_{n}=0$$

Using the orthogonal method and integrating the equations from 0 to 1 yields the following:

(18) $$\xi_{0n}^{**}+\frac{\int_{0}^{1}\phi_{n}^{iv}\phi_{n}d\bar{x}}{\int_{0}^{1}\phi_{n}\phi_{n}d\bar{x}-{\bar{I}}_{b}\int_{0}^{1}\phi_{n}^{\prime \prime }\phi_{n}d\bar{x}}\xi_{0n}=0$$

(19) $$\begin{array}{l} \xi_{1n}^{**}+\frac{\int_{0}^{1}\phi_{n}^{iv}\phi_{n}d\bar{x}}{\int_{0}^{1}\phi_{n}\phi_{n}d\bar{x}-{\bar{I}}_{b}\int_{0}^{1}\phi_{n}^{\prime \prime }\phi_{n}d\bar{x}}\xi_{1n}=-2\frac{\partial }{\partial T_{1}}\xi_{0n}^{*}+2\frac{\partial }{\partial T_{1}}{\bar{I}}_{b}\xi_{0n}^{*}\frac{\int_{0}^{1}\phi_{n}^{\prime \prime }\phi_{n}d\bar{x}}{\int_{0}^{1}\phi_{n}\phi_{n}d\bar{x}}\\ -\frac{1}{2\int_{0}^{1}\phi_{n}\phi_{n}d\bar{x}}\xi_{0n}(\xi_{0j}^{**}\xi_{0k}+2\xi_{0j}^{*}\xi_{0k}^{*}+\xi_{0j}\xi_{0k}^{**})\\ \;(\int_{0}^{1}\phi_{n}({\phi^{\prime \prime }}_{n}\int_{1}^{\bar{x}}\int_{0}^{\bar{x}}{\phi^{\prime }}_{j}{\phi^{\prime }}_{k}d\bar{x}d\bar{x}+{\phi^{\prime }}_{n}(\int_{1}^{\bar{x}}\int_{0}^{\bar{x}}{\phi^{\prime }}_{j}{\phi^{\prime }}_{k}d\bar{x}d\bar{x}{)}^{\prime })d\bar{x})-\mu_{w}\xi_{0n}^{*}+{\bar{q}}_{n}\frac{\int_{0}^{1}\phi_{n}d\bar{x}}{\int_{0}^{1}\phi_{n}\phi_{n}d\bar{x}} \end{array}$$

From Equation (18), the natural frequencies of this system are derived as follows:

(20) $$\omega_{n}={\left[\frac{\int_{0}^{1}\phi_{n}^{iv}\phi_{n}d\bar{x}}{\int_{0}^{1}\phi_{n}\phi_{n}d\bar{x}-{\bar{I}}_{b}\int_{0}^{1}\phi_{n}^{\prime \prime }\phi_{n}^{\prime \prime }\phi_{n}d\bar{x}}\right]}^{\frac{1}{2}}$$

To examine whether internal resonance combinations can be achieved in this system, we fixed the length of an elastic steel and assumed three circular plates of different diameters (dimensionless diameters of 0.4, 0.8, and 1.2) made of steel. Based on this, we calculated the corresponding dimensionless moment of inertia ${\bar{I}}_{b}$ of the elastic beam for each plate diameter using Equation (20). By keeping the material and length of the elastic steel constant and adjusting the beam’s cross-sectional width and thickness, we modified its mass to achieve an appropriate ${\bar{I}}_{b}$ value, such that the system’s first and third natural frequencies satisfy a 1:3 internal resonance condition. This internal energy transfer mechanism allows vibrations from higher modes to shift into the low-frequency mode, thereby enabling more effective excitation and energy harvesting through low-frequency responses. Table 1 lists the ${\bar{I}}_{b}$ values and natural frequencies corresponding to the three plate diameters.

### 2.3. Frequency Response Analysis

From Equation (18), the time-dependent general solution can be expressed as follows:

(21) $$\xi_{0n}\left(\tau \right)=B_{n}\left(T_{1}\right)e^{-i\zeta_{n}}e^{i\omega_{n}T_{0}}+{\bar{B}}_{n}\left(T_{1}\right)e^{i\zeta_{n}}e^{-i\omega_{n}T_{0}}$$

where ζ is the phase angle, and *B_n_* is the amplitude of the *n*^th^ mode.

Differentiating Equation (21) with respect to time, substituting the result into Equation (19), and assuming the external force follows simple harmonic motion, $\bar{q}$ can be represented in the following form:

(22) $${\bar{q}}_{n}={\bar{q}}_{n}e^{i\Omega \tau }={\bar{q}}_{n}e^{i\left(\omega_{n}+\varepsilon^{2}\sigma \right)T_{0}}={\bar{q}}_{n}\left(e^{i\varepsilon^{2}\sigma T_{0}}e^{i\omega_{n}T_{0}}\right)={\bar{q}}_{n}e^{i\sigma T_{1}}e^{i\omega_{n}T_{0}}$$

where Ω is the frequency of the external force, and σ is the tuning frequency near the natural frequency. Considering the internal resonance phenomenon in Equation (20), we analyze only the coupling between the first mode and the third mode. To obtain the Solvability Condition, the secular terms must be extracted and set to zero. The secular terms for the first mode are all terms containing $e^{i\omega_{1}T_{0}}$ and $e^{i\omega_{3}T_{0}-2i\omega_{1}T_{0}}$, as follows:

(23) $$\begin{array}{l} -2\left(i\omega_{1}B_{1}^{\prime }e^{-i\zeta_{1}}+\omega_{1}\zeta_{1}^{\prime }B_{1}e^{-i\zeta_{1}}\right)+2{\bar{I}}_{b}\left(i\omega_{1}B_{1}^{\prime }e^{-i\zeta_{1}}+\omega_{1}\zeta_{1}^{\prime }B_{1}e^{-i\zeta_{1}}\right)\frac{\int_{0}^{1}\phi_{1}^{\prime \prime }\phi_{1}d\bar{x}}{\int_{0}^{1}\phi_{1}^{2}d\bar{x}}\;+\omega_{1}^{2}\left(\frac{3}{2}B_{1}^{2}{\bar{B}}_{1}e^{-i\zeta_{1}}\frac{\int_{0}^{1}\phi_{1}^{\prime \prime }\phi_{1}^{\prime 2}\phi_{1}d\bar{x}}{\int_{0}^{1}\phi_{1}^{2}d\bar{x}}\right.\\ \;+{\bar{B}}_{1}^{2}B_{3}e^{2i\zeta_{1}-i\zeta_{3}}\frac{\int_{0}^{1}\phi_{1}^{\prime \prime }\phi_{1}^{\prime }\phi_{3}^{\prime }\phi_{1}d\bar{x}}{\int_{0}^{1}\phi_{1}^{2}d\bar{x}}\;+B_{1}B_{3}{\bar{B}}_{3}e^{-\zeta_{1}}\frac{\int_{0}^{1}\phi_{1}^{\prime \prime }\phi_{3}^{\prime 2}\phi_{1}d\bar{x}}{\int_{0}^{1}\phi_{1}^{2}d\bar{x}}+\frac{1}{2}{\bar{B}}_{1}^{2}B_{3}e^{2i\zeta_{1}-i\zeta_{3}}\frac{\int_{0}^{1}\phi_{3}^{\prime \prime }\phi_{1}^{\prime 2}\phi_{1}d\bar{x}}{\int_{0}^{1}\phi_{3}\phi_{1}d\bar{x}}\;\left.+2B_{1}B_{3}{\bar{B}}_{3}e^{-i\zeta_{1}}\frac{\int_{0}^{1}\phi_{3}^{\prime \prime }\phi_{1}^{\prime }\phi_{3}^{\prime }\phi_{1}d\bar{x}}{\int_{0}^{1}\phi_{3}\phi_{1}d\bar{x}}\right)\\ \;-\mu_{w}i\omega_{1}B_{1}e^{-i\zeta_{1}}+{\bar{q}}_{1}e^{i\sigma T_{1}}\frac{\int_{0}^{1}\phi_{1}d\bar{x}}{\int_{0}^{1}\phi_{1}^{2}d\bar{x}} \end{array}$$

The secular terms for the third mode are obtained by extracting all terms containing $e^{i\omega_{3}T_{0}}$ and $e^{3i\omega_{1}T_{0}}$ as follows:

(24) $$\begin{array}{l} -2\left(i\omega_{3}B_{3}^{\prime }e^{-i\zeta_{3}}+\omega_{3}\zeta_{3}^{\prime }B_{3}e^{-i\zeta_{3}}\right)+2{\bar{I}}_{b}\left(i\omega_{3}B_{3}^{\prime }e^{-i\zeta_{3}}+\omega_{3}\zeta_{3}^{\prime }B_{3}e^{-i\zeta_{3}}\right)\frac{\int_{0}^{1}\phi_{3}^{\prime \prime }\phi_{3}d\bar{x}}{\int_{0}^{1}\phi_{3}^{2}d\bar{x}}\;+\omega_{3}^{2}\left(\frac{1}{2}B_{1}^{3}e^{-3i\zeta_{1}}\frac{\int_{0}^{1}\phi_{1}^{\prime \prime }\phi_{n}^{\prime \prime }\phi_{1}^{\prime }\phi_{1}^{\prime }\phi_{3}d\bar{x}}{\int_{0}^{1}\phi_{1}\phi_{3}d\bar{x}}\right.\\ \;+2B_{1}{\bar{B}}_{1}B_{3}e^{-i\zeta_{3}}\frac{\int_{0}^{1}\phi_{1}^{\prime \prime }\phi_{1}^{\prime }\phi_{3}^{\prime }\phi_{3}d\bar{x}}{\int_{0}^{1}\phi_{1}\phi_{3}d\bar{x}}\;\left.+B_{1}{\bar{B}}_{1}B_{3}e^{-i\zeta_{3}}\frac{\int_{0}^{1}\phi_{3}^{\prime \prime }\phi_{1}^{\prime }\phi_{1}^{\prime }\phi_{3}d\bar{x}}{\int_{0}^{1}\phi_{3}^{2}d\bar{x}}+\frac{3}{2}B_{3}^{2}{\bar{B}}_{3}e^{-i\zeta_{3}}e^{i\omega_{3}T_{0}}\frac{\int_{0}^{1}\phi_{3}^{\prime \prime }\phi_{3}^{\prime }\phi_{3}^{\prime }\phi_{3}d\bar{x}}{\int_{0}^{1}\phi_{3}\phi_{3}d\bar{x}}\right)\\ \;-\mu_{w}i\omega_{3}B_{3}e^{-i\zeta_{3}}+{\bar{q}}_{3}e^{i\sigma T_{1}}\frac{\int_{0}^{1}\phi_{3}d\bar{x}}{\int_{0}^{1}\phi_{3}^{2}d\bar{x}} \end{array}$$

For brevity and improved readability, detailed analytical steps related to the internal resonance response and stability analysis have been moved to Appendix B. In this appendix, the perturbation expansion and solvability conditions are developed to derive the steady-state amplitude and phase relations for the first and third vibration modes. The resulting expressions form the basis for constructing the fixed-point diagrams and frequency–response curves discussed in the following sections.

**Figure 5:**
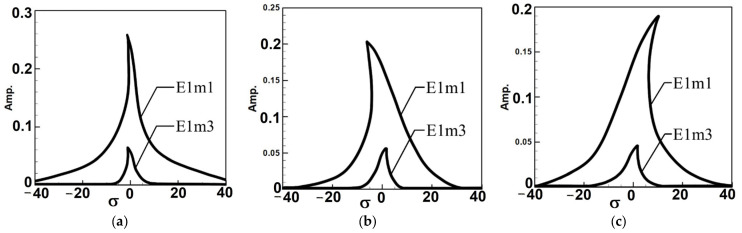
Fixed-point plots of the BIS-VEHS system under first-mode excitation: (**a**) diameter ratio = 0.4, (**b**) diameter ratio = 0.8, and (**c**) diameter ratio = 1.2. As the diameter ratio increases, the fixed-point spacing and amplitude level broaden, indicating stronger modal coupling and enhanced energy transfer potential under internal resonance conditions.

To analyze the system’s frequency response and make the fixed-points plots, Equations (A9)–(A11) were derived using the orthogonal method by multiplying each equation by $\phi_{1}$ and integrating from 0 to 1 to decouple the equations. The fixed-point plots for the first and third modes under the first mode excitation are shown in Figure 5. Where E1m1 represents the first mode fixed-point plot under first-mode excitation, E1m3 represents the third mode fixed-point plot under first-mode excitation. The fixed points illustrate how increasing the circular-plate diameter alters the equilibrium amplitude and stability of the nonlinear system. As the diameter ratio increases, the fixed-point spacing and amplitude level broaden, indicating stronger modal coupling and enhanced energy transfer potential under internal resonance conditions.

**Figure 6:**
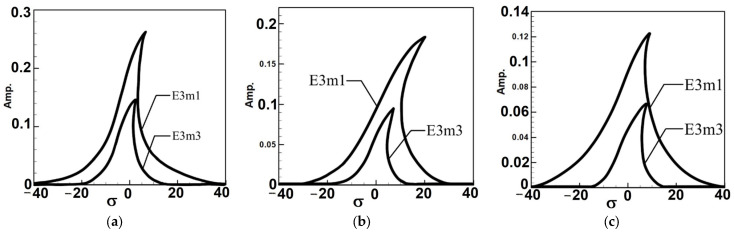
Fixed-point plots of the BIS-VEHS system under third-mode excitation: (**a**) diameter ratio = 0.4, (**b**) diameter ratio = 0.8, and (**c**) diameter ratio = 1.2. It can be seen that when the third mode is excited, the amplitude of the first mode becomes significantly larger, indicating the occurrence of internal resonance.

Next, consider the case of exciting the third mode. Since this corresponds to third-mode excitation, the external force on the first mode is zero. Due to space constraints and methodological similarity to the first mode analysis, the results are presented directly as fixed-point plots. These plots depict the amplitudes of the first and third modes under third mode excitation, as shown in Figure 6. Here, E3m1 and E3m3 represent the fixed-point plots of the first and third modes, respectively, under third-mode excitation.

Observing Figure 5 and Figure 6, which depict the amplitudes of the first and third modes under their respective excitations, it can be seen from Figure 6 that when the third mode is excited, the amplitude of the first mode is significantly larger. This indicates the occurrence of internal resonance, wherein the energy transfer from higher modes results in greater amplitudes in lower modes. This confirms that the system exhibits internal resonance behavior. Table 2 shows the maximum amplitudes for three different circular plate diameter ratios (D) in a system with internal resonance under excitation of the first and third modes.

**Figure 7:**
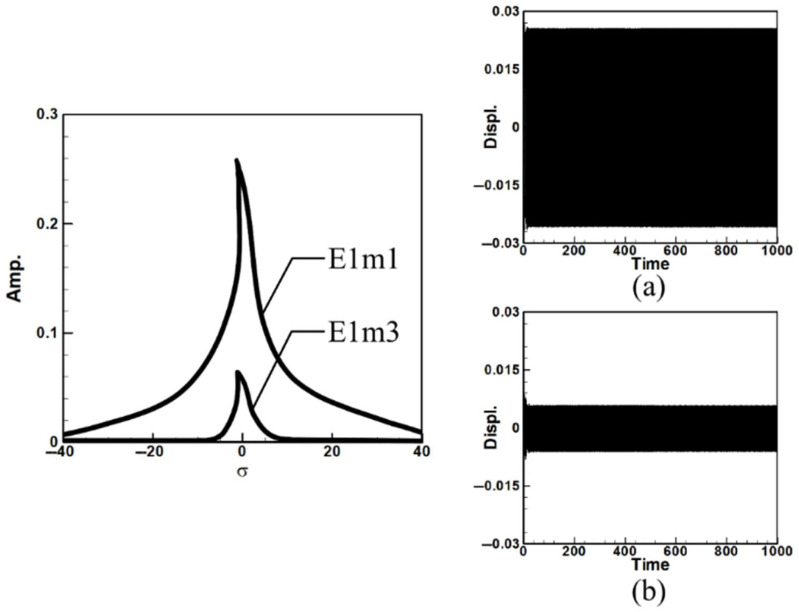
Dimensionless fixed-point and time response plots under first-mode excitation for a circular plate with a diameter ratio of 0.4: (**a**) E1m1, (**b**) E1m3.

**Figure 8:**
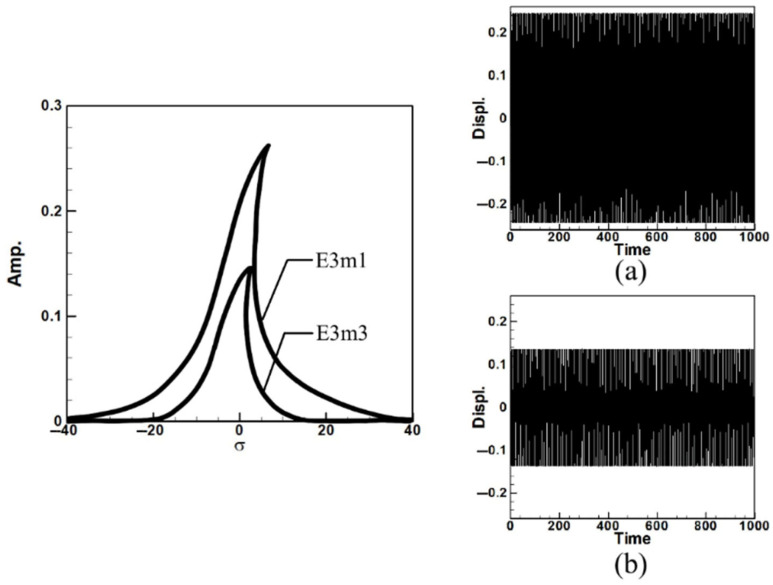
Dimensionless fixed-point and time response plots under third mode excitation, the diameter ratio of the circular plate is 0.4: (**a**) E3m1, (**b**) E3m3.

**Figure 9:**
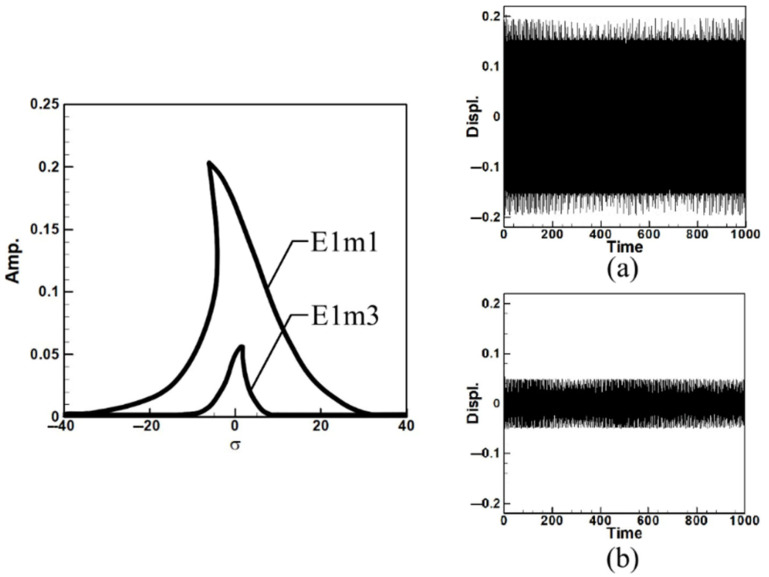
Dimensionless fixed-points and time response plots under first mode excitation, the diameter ratio of the circular plate is 0.8: (**a**) E1m1, (**b**) E1m3.

**Figure 10:**
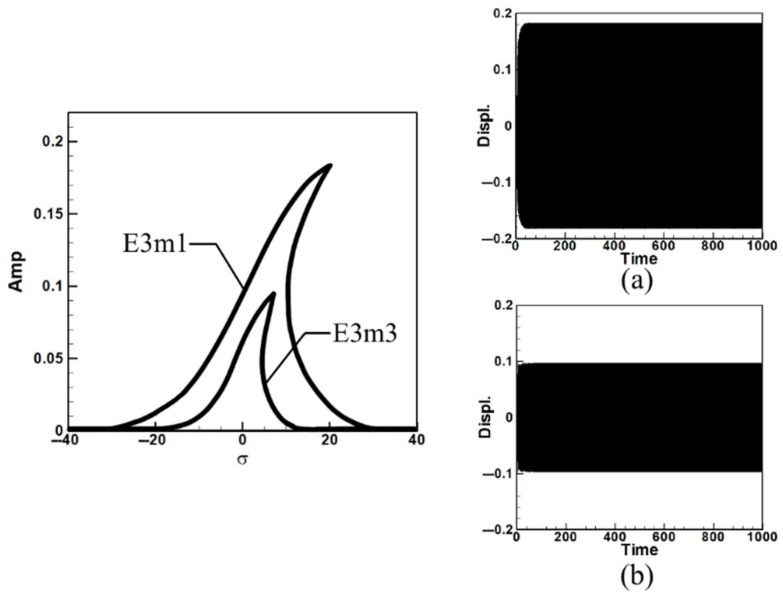
Dimensionless fixed-points and time response plots under third mode excitation, the diameter ratio of the circular plate is 0.8: (**a**) E3m1, (**b**) E3m3.

**Figure 11:**
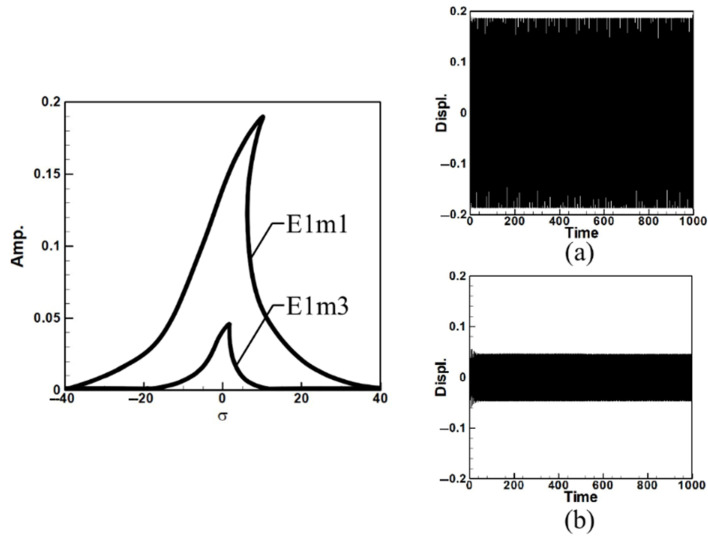
Dimensionless fixed-points and time response plots under first mode excitation, the diameter ratio of the circular plate is 1.2: (**a**) E1m1, (**b**) E1m3.

**Figure 12:**
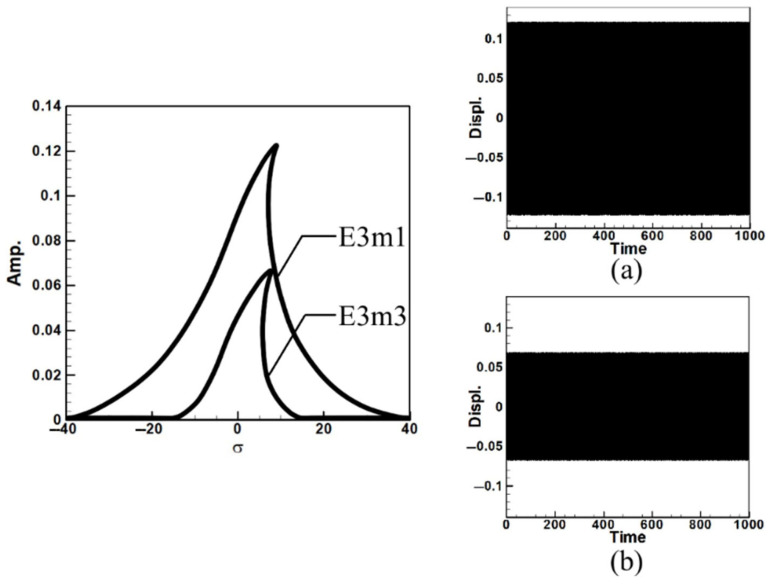
Dimensionless fixed-points and time response plots under third mode excitation, the diameter ratio of the circular plate is 1.2: (**a**) E3m1, (**b**) E3m3.

Next, the time response plots of the amplitudes were generated using the fourth-order Runge–Kutta (RK-4) numerical method to verify the accuracy of the maximum amplitudes obtained from the fixed-points plot. Figure 7, Figure 8, Figure 9, Figure 10, Figure 11 and Figure 12 show the time response plots corresponding to the first and third mode excitations for the three different circular plate diameters in the system with internal resonance. From Figure 7, Figure 8, Figure 9, Figure 10, Figure 11 and Figure 12, it can be observed that the beam displacements obtained using the RK-4 method are consistent with those predicted by the fixed-points plot, confirming the presence of internal resonance in the system. 

**Figure 13:**
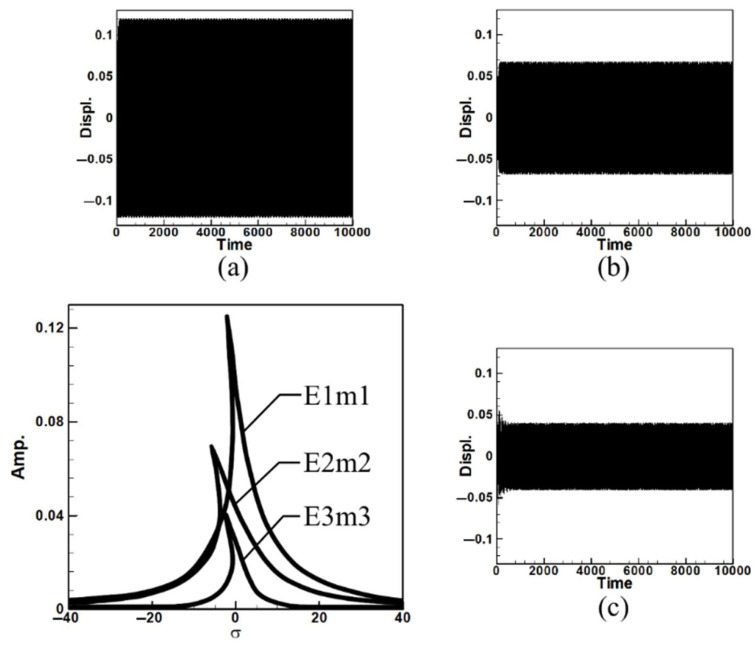
Dimensionless fixed-point and time response plots under first to third mode excitation for a circular plate with a diameter ratio of 0.4: (**a**) E1m1, (**b**) E2m2, (**c**) E3m3.

**Figure 14:**
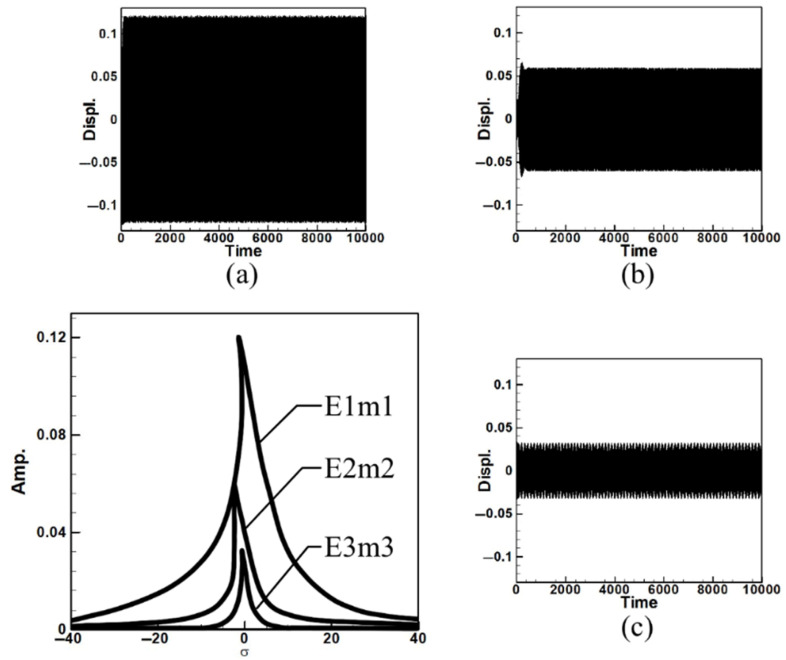
Dimensionless fixed-point and time response plots under first to third mode excitation for a circular plate with a diameter ratio of 0.8: (**a**) E1m1, (**b**) E2m2, (**c**) E3m3.

**Figure 15:**
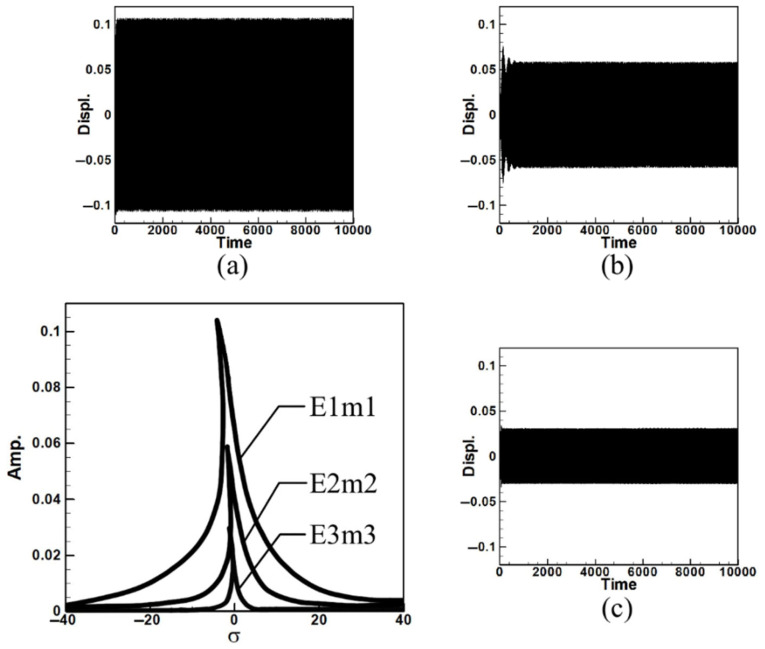
Dimensionless fixed-point and time response plots under first to third mode excitation for a circular plate with a diameter ratio of 1.2: (**a**) E1m1, (**b**) E2m2, (**c**) E3m3.

Since internal resonance is uncommon in real-world scenarios, this study also considers cases without internal resonance. Figure 13, Figure 14 and Figure 15 show the fixed-point plots and corresponding time response plots for circular plates with dimensionless diameters of 0.4, 0.8, and 1.2 under excitation of the first three modes in the absence of internal resonance.

Without internal resonance, the response is limited to the excited mode only. That is, when the first, second, or third mode is individually excited, only the corresponding mode exhibits a significant response, while the amplitudes of the non-excited modes are negligible. Table 3 presents the maximum amplitudes for the three circular plate diameters under excitation of the first three modes in the non-resonant system.

It is evident from Figure 13, Figure 14 and Figure 15 and Table 3 that the time response plots generated using RK-4 are consistent with the fixed-points plots, thereby enabling the next step of electrical energy conversion analysis.

### 2.4. Piezoelectric Equations

In this study, a PZT is placed at the root of the elastic steel beam. Based on the research by Rajora et al. [26], the piezoelectric equations for the PZT can be expressed as follows:

(25) $$C_{p}\dot{V}+\frac{1}{R_{p}}V+\int_{a}^{b}eh_{p}t_{h}{\dot{w}}^{\prime \prime }dx=0$$

where *V* is the voltage, *C_p_* is the capacitance of the piezoelectric patch, *R_p_* denotes the load resistance, *e* stands for the dielectric constant, *h_p_* represents the length of the PZT, and *t_h_* is the thickness of the PZT. The Coulomb force exerted by the PZT on this nonlinear beam can be expressed as follows:

(26) $$\int_{a}^{b}eh_{p}t_{h}{\dot{w}}^{\prime \prime }dx\frac{V}{h_{p}}=\left(et_{h}\int_{a}^{b}{\dot{w}}^{\prime \prime }dx\right)V=C_{f}\left(\int_{a}^{b}{\dot{w}}^{\prime \prime }dx\right)V$$

Here, *C_p_* represents the capacitance of the PZT, and *C_f_* is the piezoelectric coupling coefficient. The dimensionless piezoelectric equation for the PZT can be expressed as follows:

(27) $${\bar{v}}^{*}+{\bar{R}}_{p}v+\hat{k}\int_{\bar{a}}^{\bar{b}}{\left({\bar{w}}^{\prime \prime }\right)}^{*}d\bar{x}=0$$

Here, $\bar{v}=\frac{V}{C_{f}}$, ${\bar{R}}_{p}=\frac{1}{R_{p}C_{p}\omega }$, $\hat{k}=\frac{eh_{p}t_{h}}{C_{p}C_{f}}$. Substituting into Equation (27) and processing yields

(28) $$\bar{v}=-\frac{\hat{k}}{e^{{\bar{R}}_{p}\tau }}\int_{0}^{\tau }\left[\int_{\bar{a}}^{\bar{b}}{\left({\bar{w}}^{\prime \prime }\right)}^{*}d\bar{x}\right]e^{{\bar{R}}_{p}\tau }d\tau$$

Thus, the dimensionless piezoelectric equation can be written as follows:

(29) $$\frac{C_{f}^{2}(\int_{\bar{a}}^{\bar{b}}{\bar{w}}^{\prime \prime }d\bar{x})\bar{v}}{lm\omega^{2}}=\frac{\hat{k}\eta^{2}}{e^{\bar{R}p\tau }}\int_{\bar{a}}^{\bar{b}}{\bar{w}}^{\prime \prime }d\bar{x}\int_{0}^{\tau }\left[\int_{\bar{a}}^{\bar{b}}{\left({\bar{w}}^{\prime \prime }\right)}^{*}d\bar{x}\right]e^{{\bar{R}}_{p}\tau }d\tau$$

In Equation (29), $\bar{a}$ and $\bar{b}$ represent the positions of the two ends of the PZT along the beam. Here, it is assumed that the length of the PZT is one-tenth of the beam’s length and is installed at the root of the beam. Thus, $\bar{a}$ and $\bar{b}$ are 0 and 0.1, respectively. Next, by setting $\eta^{2}=C_{f}^{2}/lm\omega^{2}$ and substituting into the beam equation and piezoelectric equation, the dimensionless nonlinear beam equation with PZT can be expressed as follows:

(30) $${\bar{w}}^{**}-{\bar{I}}_{b}\frac{\partial^{2}{\bar{w}}^{**}}{\partial {\bar{x}}^{2}}+\frac{\partial^{4}\bar{w}}{\partial {\bar{x}}^{4}}+\frac{1}{2}({\bar{w}}^{\prime }\int_{1}^{\bar{x}}(\int_{0}^{\bar{x}}{{\bar{w}}^{\prime }}^{2}d\bar{x}{)}^{**}d\bar{x}{)}^{\prime }-\bar{q}e^{i\Omega \tau }+\mu_{w}w^{*}\;\;-\frac{\hat{k}\eta^{2}}{e^{{\bar{R}}_{p}\tau }}\int_{\bar{a}}^{\bar{b}}{\bar{w}}^{\prime \prime }d\bar{x}\int_{0}^{\tau }\left[\int_{\bar{a}}^{\bar{b}}{\left({\bar{w}}^{\prime \prime }\right)}^{*}d\bar{x}\right]e^{{\bar{R}}_{p}\tau }d\tau =0\cdots$$

### 2.5. Simulation of Slapping Force

**Figure 16:**
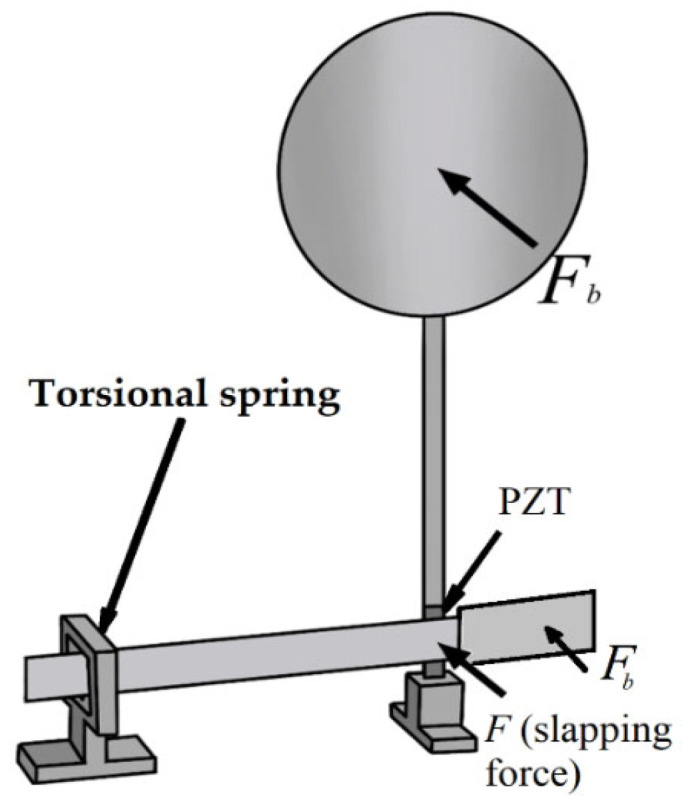
BIS-VEHS structural diagram, where *F* represents the slapping force (acting on the root of the main structure), and *F_b_* represents the wind force acting on both the circular plate and the rectangular plate.

As shown in Figure 16, the slapping force exerted by the transverse long-plate lever mechanism of this bio-inspired energy harvesting system on the PZT can be expressed as follows:

(31) $$F=\rho A{\ddot{w}}_{F}\delta (t-T),\;t>0$$

where *T* is the slapping period and $\delta \left(t\right)$ is the Dirac delta function. It is noted that Equation (31) represents the dimensional slapping excitation force *F*(*t*) applied to the system. For subsequent theoretical analysis, this force is nondimensionalized by dividing Equation (31) by $\frac{EI}{\rho Al^{3}}$, resulting in the following dimensionless slapping force:

(32) $$\bar{F}={\bar{w}}_{F}^{**}\delta (\tau -\bar{T})$$

In Equation (32), the superscript $(\cdot {)}^{*}$ denotes differentiation with respect to the nondimensional time τ. This distinction clarifies why Equations (31) and (32) appear different in form. By substituting Equation (32) into the nondimensionalized beam equation, the governing nonlinear vibration equation with slapping excitation can be obtained.

(33) $${\bar{w}}^{**}-{\bar{I}}_{b}\frac{\partial^{2}{\bar{w}}^{**}}{\partial {\bar{x}}^{2}}+\frac{\partial^{4}\bar{w}}{\partial {\bar{x}}^{4}}+\frac{1}{2}({\bar{w}}^{\prime }\int_{1}^{\bar{x}}(\int_{0}^{\bar{x}}{{\bar{w}}^{\prime }}^{2}d\bar{x}{)}^{**}d\bar{x}{)}^{\prime }-\bar{q}e^{i\Omega \tau }+\mu_{w}{\bar{w}}^{*}\;-\frac{\hat{k}\eta^{2}}{e^{{\bar{R}}_{p}\tau }}\int_{a}^{b}{\bar{w}}^{\prime \prime }d\bar{x}\int_{0}^{\tau }\left[\int_{\bar{a}}^{\bar{b}}{\left({\bar{w}}^{\prime \prime }\right)}^{*}d\bar{x}\right]e^{{\bar{R}}_{p}\tau }d\tau =F_{b}+{\bar{w}}_{F}^{**}\delta (\tau -\bar{T})$$

where *F_b_* is the external force that follows simple harmonic motion, $\bar{q}$, as shown in Equation (22). As shown in Equation (33), the theoretical model for the Bio-inspired Epiphytic Plant Slapping Vibration Energy Harvesting System (BIS-VEHS) is now complete.

## 3. Theoretical Energy Harvesting Efficiency Analysis

**Figure 17:**
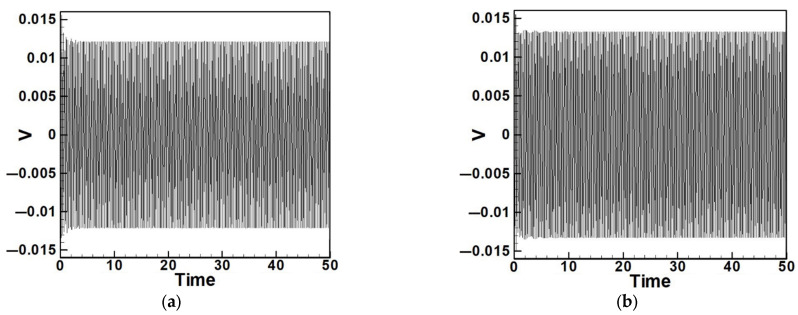

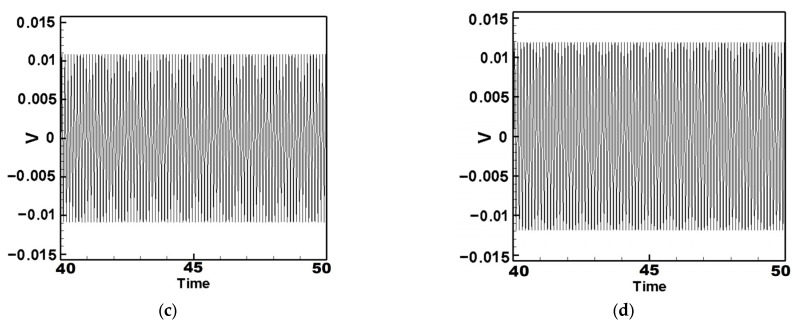
Time response of the first mode under first-mode excitation for a circular plate with a diameter ratio of 0.4 (**a**) Without slapping force, (**b**) with slapping force, (**c**) zoom out of Figure (**a**), (**d**) zoom out of Figure (**b**).

**Figure 18:**
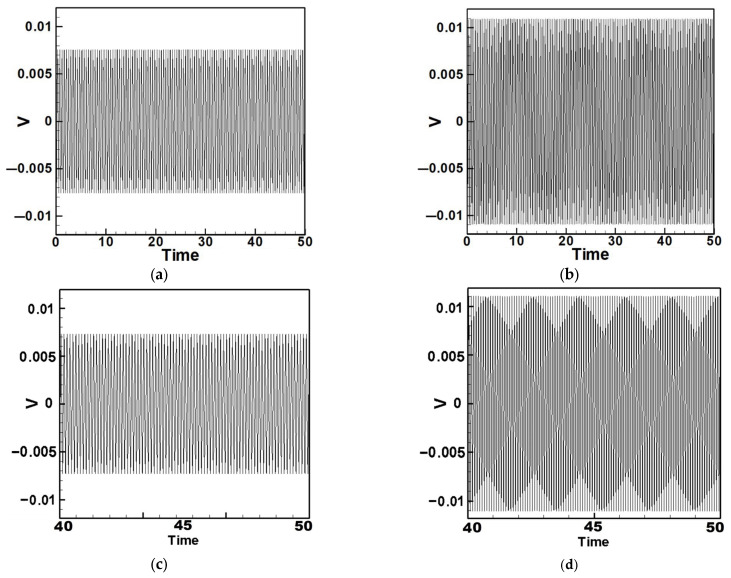
Time response of the third mode under third-mode excitation for a circular plate with a diameter ratio of 0.4. (**a**) Without slapping force, (**b**) with slapping force, (**c**) zoom out of Figure (**a**), (**d**) zoom out of Figure (**b**).

**Figure 19:**
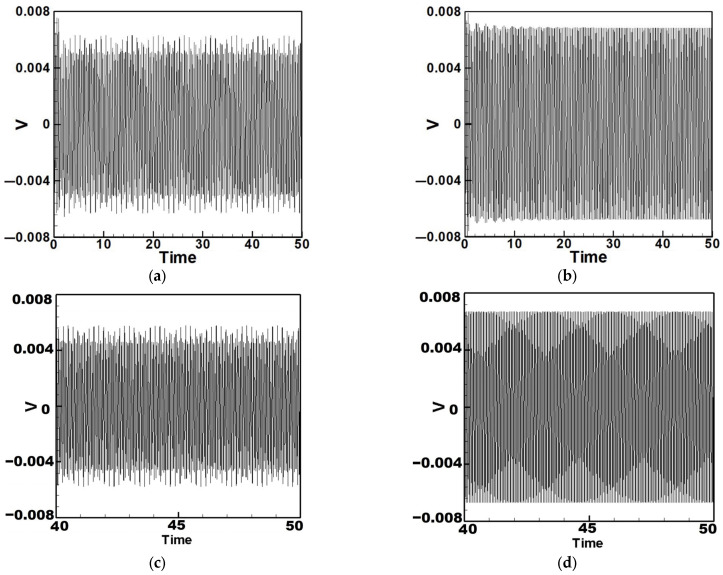
Time response of the first mode under first-mode excitation for a circular plate with a diameter ratio of 0.8. (**a**) Without slapping force, (**b**) with slapping force, (**c**) zoom out of Figure (**a**), (**d**) zoom out of Figure (**b**).

**Figure 20:**
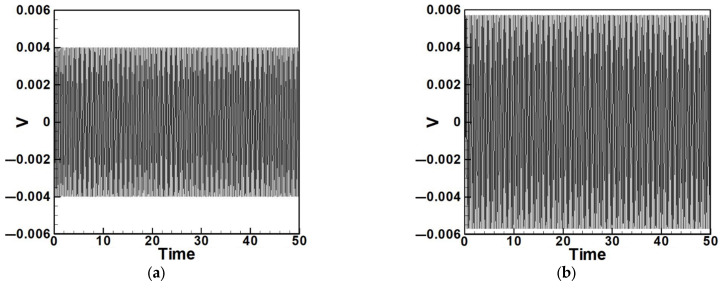

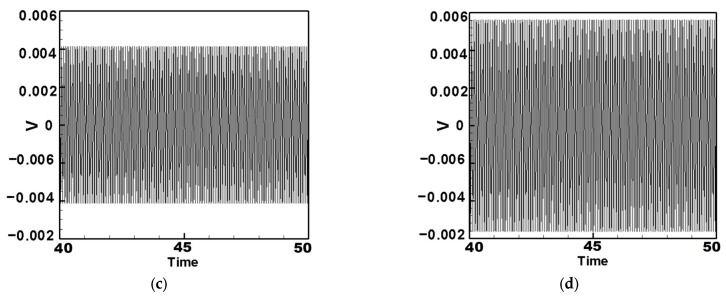
Time response of the third mode under third-mode excitation for a circular plate with a diameter ratio of 0.8. (**a**) Without slapping force, (**b**) with slapping force, (**c**) zoom out of Figure (**a**), (**d**) zoom out of Figure (**b**).

**Figure 21:**
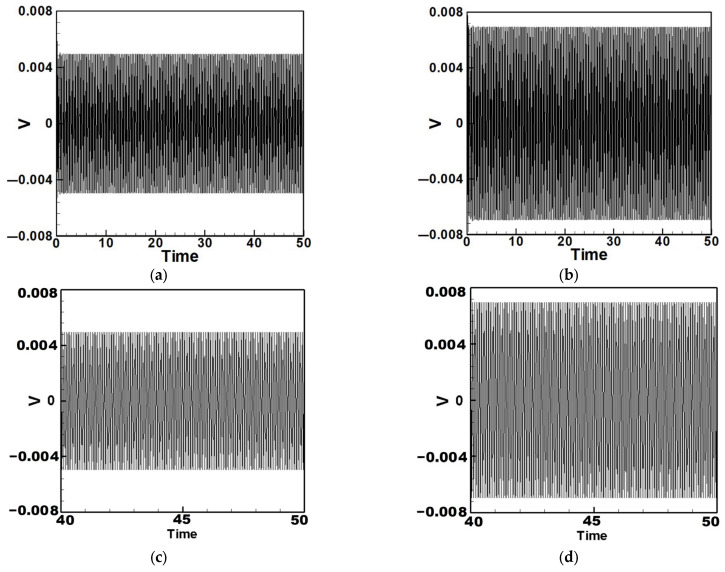
Time response of the first mode under first-mode excitation for a circular plate with a diameter ratio of 1.2. (**a**) Without slapping force, (**b**) with slapping force, (**c**) zoom out of Figure (**a**), (**d**) zoom out of Figure (**b**).

**Figure 22:**
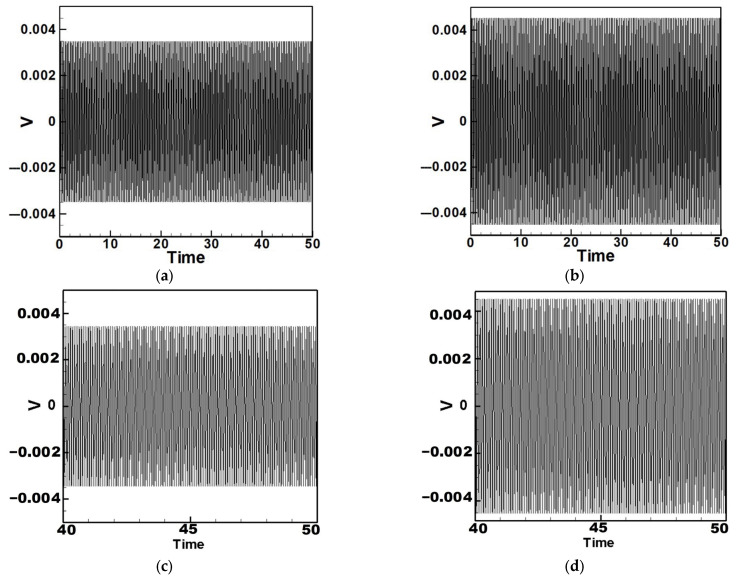
Time response of the third mode under third-mode excitation for a circular plate with a diameter ratio of 1.2. (**a**) Without slapping force, (**b**) with slapping force, (**c**) zoom out of Figure (**a**), (**d**) zoom out of Figure (**b**).

Equation (33) was solved using the RK-4 method, and the results were substituted into Equation (28) to generate voltage–time response plots for the first and third modes of the BIS-VEHS system. The analysis is divided into two cases: one without a slapping force and one with a slapping force, allowing for a comparison of energy harvesting efficiency. In the absence of a slapping force, only the primary structure’s vibrations due to external excitation are considered, without any additional impacts from surrounding elements. Energy conversion in this case relies solely on the oscillations of the main structure. When the slapping force is introduced, additional forces are applied at the root of the primary structure. As the main structure oscillates, the root simultaneously experiences slapping impacts, enabling energy harvesting from both the structure’s vibration and the slapping motion. Figure 17, Figure 18, Figure 19, Figure 20, Figure 21 and Figure 22 present the voltage outputs for both cases, highlighting their respective energy harvesting efficiencies.

After calculating the root mean square (RMS) values, the results are organized in Table 4. Under internal resonance conditions, the output voltage for the three different plate diameters is compared. Table 4 indicates that, due to the presence of internal resonance, the first mode consistently produces a higher output voltage, regardless of whether the first or third mode is excited. Moreover, the output voltage is significantly greater when the slapping force is applied compared to when it is absent, validating the theoretical assumptions proposed earlier.

In addition to the internal resonance case, the non-resonant condition is also considered. Based on the non-resonant scenario, the small perturbation equations were solved using the fourth-order Runge–Kutta method. The output voltages for the three circular plate diameter ratios were plotted under excitation of the first three modes in the absence of internal resonance. Table 5 presents the output voltages with and without the slapping force for each of the three diameter ratios under the first three mode excitations.

From Table 4 and Table 5, it can be observed that the addition of the slapping force significantly enhances the output voltage across all cases. It is also evident that as the diameter of the circular plate increases, both the beam amplitude and the output voltage gradually decrease. This is because a larger moment of inertia makes it more difficult to induce motion under the same external force. These results confirm the validity of the theoretical assumptions proposed earlier. These results can also be compared with those under internal resonance conditions to determine which scenario achieves better energy harvesting efficiency. Additionally, the theoretical predictions will be further validated through comparison with experimental results.

## 4. Experimental Setup

Building on the theoretical analysis, this study employed the multi-scale method to generate fixed-point plots, time response plots, and theoretical energy harvesting efficiency charts. The results confirmed the presence of internal resonance in the system and demonstrated that incorporating the slapping force enhances voltage output compared to the system without it. The following section details the experimental procedures designed to validate the accuracy and feasibility of the theoretical analysis.

### 4.1. Experimental Model

**Figure 23:**
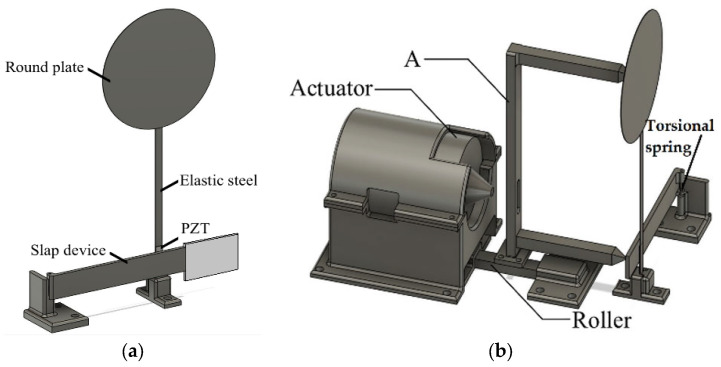
Conceptual schematic of the BIS-VEHS experimental setup: (**a**) overall system configuration; (**b**) rail-mounted linkage device (Device A), which enables synchronized excitation of both the circular plate and the bottom-slapping mechanism by the actuator.

In accordance with the theoretical model, the experimental setup was designed to replicate the structure and dynamic behavior of an epiphytic plant. It comprises four key components: an elastic steel beam, a circular plate, a piezoelectric patch (PZT-5H, procured from Steminc, Steiner and Martins, Inc., Doral, FL, USA), and a rigid slapping mechanism, as illustrated in Figure 23a. The elastic steel beam simulates the plant’s stem and serves as the primary structural element, clamped at one end with the other end free to oscillate. A circular plate, representing the plant’s leaves, is attached to the free end of the beam. Based on the diameter of the circular plate, its thickness is adjusted to ensure that the resulting mass meets the modal resonance condition. A piezoelectric patch (PZT) is installed at the root of the beam to convert mechanical vibrations into electrical energy. Finally, a rigid long-plate slapping mechanism is incorporated to simulate the natural slapping motion at the base of a plant, striking the PZT to further enhance power generation efficiency. This bio-inspired experimental configuration enables systematic evaluation of the system’s dynamic response and validation of the theoretical predictions under various excitation conditions. In the experimental setup of this study, an actuator (vibration exciter) is used to apply external disturbances, simulating vibrations induced by natural forces such as wind or other external influences, as shown in Figure 23b. The actuator is strategically positioned to excite both the circular plate and the bottom-slapping mechanism. To achieve simultaneous excitation of these two components, a linkage device (labeled as Device A in Figure 23b) has been specifically designed. The actuator is mounted on this linkage device, which is placed on a rail, ensuring that both the circular plate and the bottom-slapping mechanism experience synchronized disturbances. The bottom-slapping mechanism installed in this VEH system is fixed to the base at one end using a torsional spring, allowing the other end to swing back and forth after receiving impact forces, thereby repeatedly slapping the PZT mounted at the beam root. To validate the theoretical voltage values obtained by converting vibrational energy into electrical energy, a precise voltage measurement system (imc^TM^ system, CS-5008-1, TUV Rheinland, Kolle, Germany) is employed in the experiment. This system records the output voltage data from the PZT in real-time. The theoretical analysis suggests the presence of internal resonance in the system. Therefore, a frequency response analyzer is used to measure the resonance frequencies and vibration responses of different modes at various excitation frequencies and slapping forces. This will help verify whether the system reaches the internal resonance condition as predicted in the theoretical analysis.

### 4.2. External Setup

This study investigates the Bio-Inspired Epiphytic Plant Slapping Vibration Energy Harvesting System (BIS-VEHS), which utilizes a circular plate as the primary component for experiencing external forces. The system also features a slender, elastic steel structure resembling a stem, with a PZT positioned at its base for energy conversion. Additionally, a rigid slapping mechanism synchronously strikes the PZT, enhancing energy harvesting efficiency when the system is excited by external forces. The experiment is carried out in two key stages to validate the effectiveness of the design. First, the harvesting efficiency with and without slapping forces is compared. This step highlights the superior efficiency of the system when slapping forces are applied to the PZT, in contrast to the system without slapping forces. Next, the dynamic responses of the system under internal resonance conditions are investigated. This stage examines the vibrational behavior of the elastic steel beam when internal resonance occurs and analyzes the energy converted into electricity by the PZT across different vibration modes.

#### 4.2.1. Experimental and Theoretical Validation of the System’s Natural Frequencies

**Figure 24:**
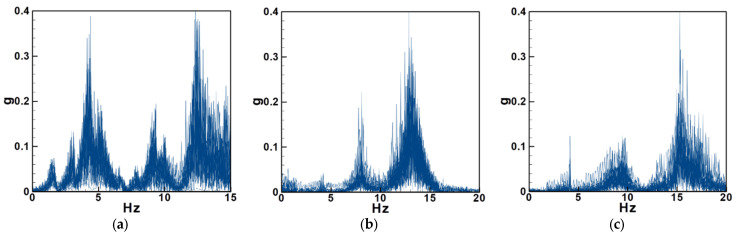
Frequency diagram of a specific circular plate and elastic steel combination, (**a**) internal resonance, (**b**) non-resonant case with circular plate diameter = 5 cm, (**c**) non-resonant case with circular plate diameter =10 cm.

In this experiment, circular plates with diameters of 5 cm and 10 cm, together with a 12.5 cm long elastic-steel beam, are employed to simulate stem-like oscillation. Experiments are conducted for systems both with internal resonance and without internal resonance, and the measured results are compared with theoretical predictions. An impact hammer and an imc™ CS-5008-1 data acquisition system are used to determine the natural frequencies of each configuration. For the internal resonance case, a 5 cm diameter circular plate (mass = 1.6 g) made of 0.1 mm thick elastic steel is attached to a 0.3 mm thick beam of 12.5 cm × 0.6 cm (mass = 2.7 g). This corresponds to the dimensional realization of the theoretical system with a dimensionless diameter of 0.4 under internal resonance. For the non-resonant system, the plate mass is adjusted using 1 mm-thick polyethylene (PE) foam boards, forming 5 cm and 10 cm plates with masses of 0.165 g and 0.66 g, respectively. The frequency responses for these cases are shown in Figure 24a–c. For completeness, the corresponding physical and dimensionless parameters used in this experimental configuration are summarized in Table A1 (Appendix C), ensuring consistency between the measured setup and the normalized simulation model. 

In the experimental setup, a vibration exciter (actuator) is employed to apply controlled external disturbances, simulating ambient vibrations such as wind or flow-induced excitation, as illustrated in Figure 23b. The actuator is mounted on a rail-guided linkage device (Device A) that allows simultaneous and synchronized excitation of both the circular plate and the bottom-slapping mechanism. This configuration ensures consistent and repeatable excitation rather than conventional base excitation. The beam is rigidly clamped at its root, providing a fixed–free boundary condition consistent with the theoretical model. To assess data reliability, the uncertainty in frequency measurement was estimated to be ±0.1 Hz, and repeated tests under identical excitation conditions exhibited less than 5% variation in RMS voltage. These results confirm the accuracy and repeatability of the measurements and validate that the experimental setup reliably represents the modeled system.

In Figure 24, the y-axis represents the dimensionless acceleration amplitude, which was used to identify the natural frequencies of the BIS-VEHS system. The acceleration values were normalized with respect to the gravitational acceleration, and for convenience, the vertical axis, labeled “g”, denotes this normalized acceleration. From Figure 24 a, it can be observed that in the internal resonance system, the low-frequency peak is higher than the high-frequency peak. This is due to internal resonance transferring energy from higher modes to lower modes. Zhang et al. [27] also noted that in systems with internal resonance, the frequency response exhibits multiple peaks. This occurs because energy is transferred within the system, and when a higher mode is excited, the lower mode is indirectly activated, resulting in multiple jump points in the response plot. The frequency diagram of the internal resonance system shows two prominent peaks at 4.25 Hz and 12.45 Hz. In contrast, the frequency responses of the non-resonant system (Figure 24b,c) show three main peaks: For the 5 cm diameter plate, these are 4.15 Hz, 8.19 Hz, and 13.15 Hz. For the 10 cm diameter plate, these are 4.07 Hz, 9.57 Hz, and 15.65 Hz. The experimental data are summarized in Table 6 and Table 7 and compared with the theoretically calculated natural frequencies.

Based on Table 6 and Table 7, the errors between the theoretical and experimental natural frequencies are all within 10%, which sufficiently validates the accuracy of the theoretical natural frequency predictions. Thus, subsequent experiments can proceed with confidence.

#### 4.2.2. Experimental and Theoretical Validation of the System’s Displacements

**Figure 25:**
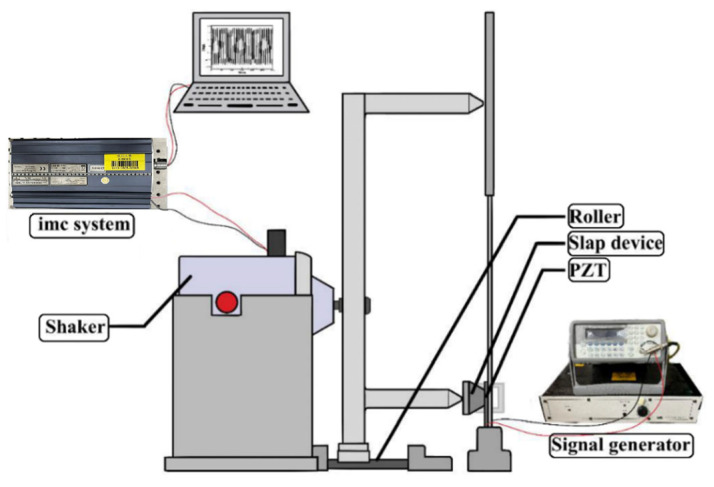
Experimental setup and system schematic diagram.

**Figure 26:**
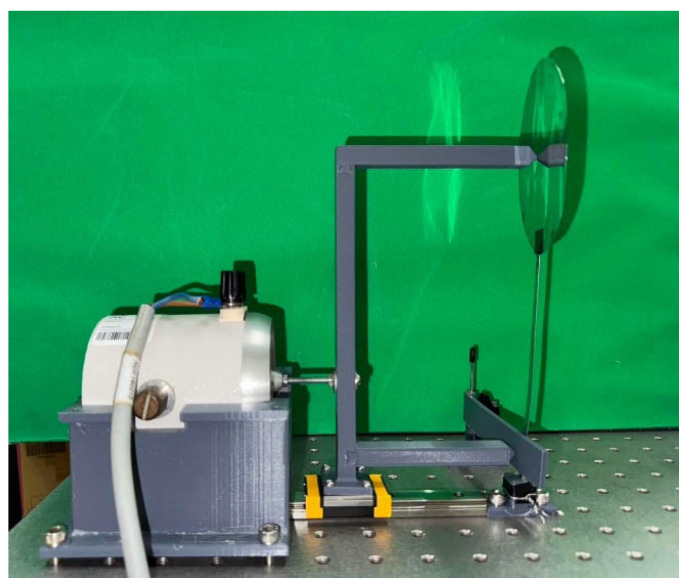
Experimental setup photo.

After assembling the elastic steel beam, circular plate, slapping mechanism, and PZT components, the entire system was mounted on a vibration platform. Careful alignment was ensured to prevent misalignment errors, allowing the actuator (vibration exciter) to accurately excite the system. The vibration exciter’s frequency was varied using a signal generator and frequency regulator. Low-to-high frequency tests were performed, particularly near the system’s primary mode frequencies, to observe excitation responses. Signals captured by the imc measurement system were analyzed through Fourier Transform to examine mode responses. The experimental setup and system schematic diagram are shown in Figure 25. The experimental setup photo is shown in Figure 26.

**Figure 27:**
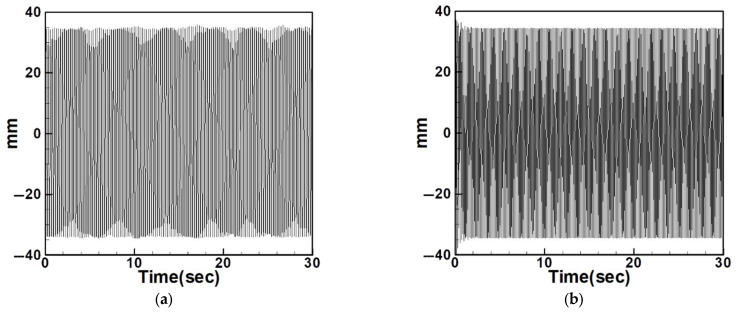
Experimental frequency–response curve of the BIS-VEHS internal resonance system under first-mode excitation, showing displacement amplitude (mm) versus excitation frequency (Hz). (**a**) Experimental displacement, (**b**) theoretical dimensional displacement.

**Figure 28:**
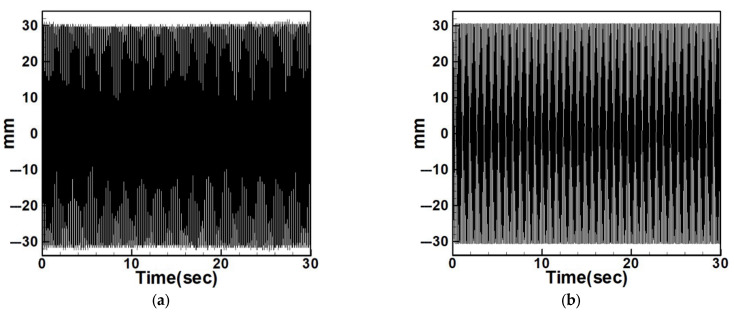
Experimental frequency–response curve of the BIS-VEHS internal resonance system under third-mode excitation, showing displacement amplitude (mm) versus excitation frequency (Hz). (**a**) Experimental displacement, (**b**) theoretical dimensional displacement.

Based on the natural frequencies calculated theoretically and measured experimentally, the system is excited using a vibration exciter to induce oscillation. The frequency of the exciter is then adjusted using a signal generator and a frequency regulator. A laser displacement sensor is used to measure the displacement of the circular plate during vibration, and the results are compared with the dimensional theoretical data. Figure 27 and Figure 28 show the comparison between experimental and dimensional theoretical displacements for the internal resonance system under first- and third-mode excitation.

**Figure 29:**
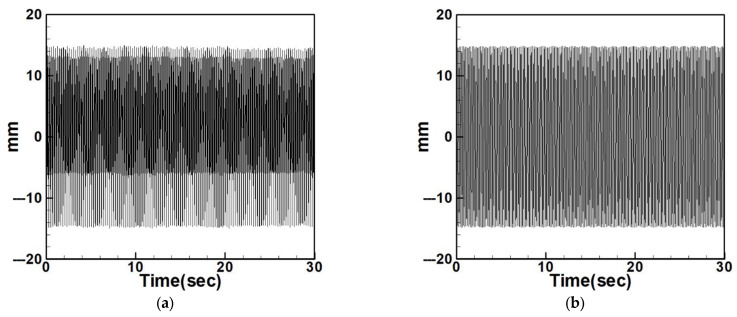
Non-resonant system with 5 cm diameter plate under first-mode excitation, (**a**) experimental displacement, (**b**) theoretical dimensional displacement.

**Figure 30:**
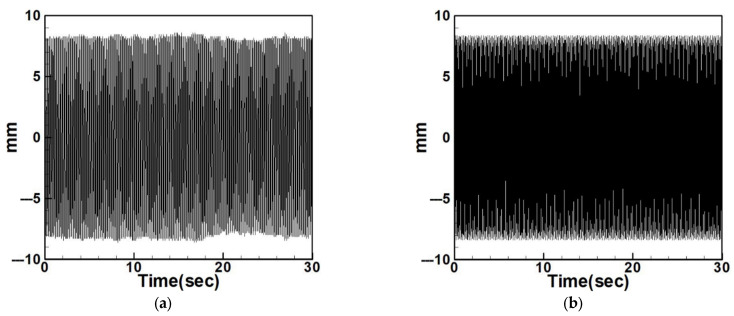
Non-resonant system with 5 cm diameter plate under second-mode excitation, (**a**) experimental displacement, (**b**) theoretical dimensional displacement.

**Figure 31:**
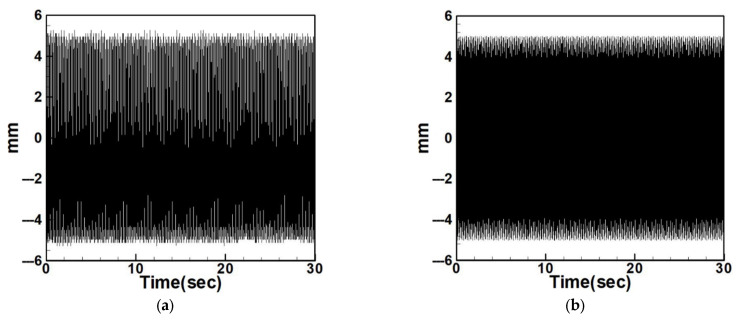
Non-resonant system with 5 cm diameter plate under third-mode excitation, (**a**) experimental displacement, (**b**) theoretical dimensional displacement.

**Figure 32:**
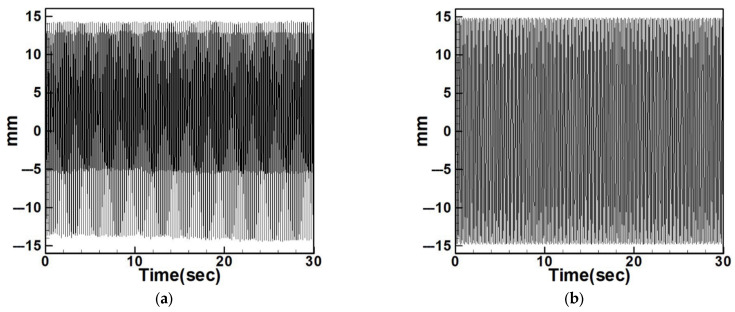
Non-resonant system with 10 cm diameter plate under first-mode excitation, (**a**) experimental displacement, (**b**) theoretical dimensional displacement.

**Figure 33:**
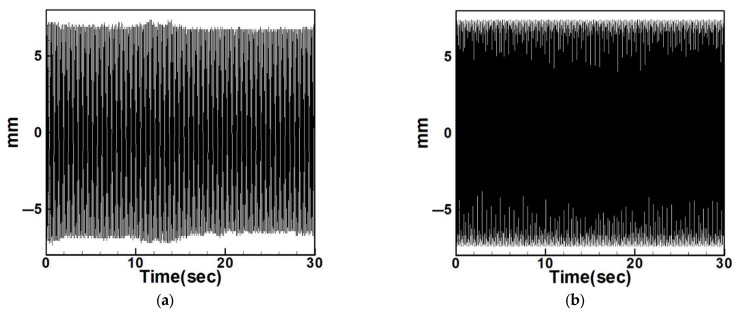
Non-resonant system with 10 cm diameter plate under second-mode excitation, (**a**) experimental displacement, (**b**) theoretical dimensional displacement.

**Figure 34:**
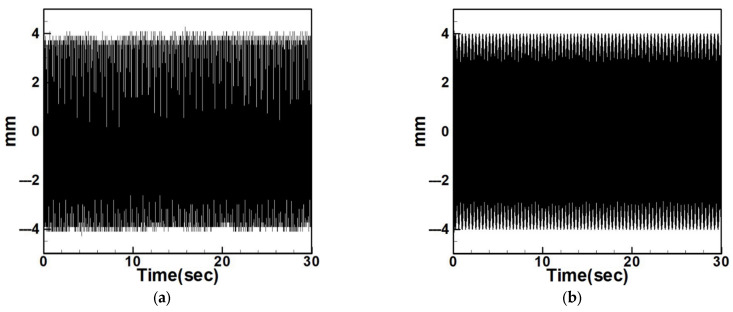
Non-resonant system with 10 cm diameter plate under third-mode excitation, (**a**) experimental displacement, (**b**) theoretical dimensional displacement.

To examine the effects of internal resonance, the study also compares the experimental displacements of the non-resonant system with its dimensional theoretical predictions. Figure 29, Figure 30, Figure 31, Figure 32, Figure 33 and Figure 34 present the displacement comparisons for non-resonant systems using 5 cm and 10 cm diameter circular plates under excitation of the first three modes.

Table 8 and Table 9 provide RMS comparisons between experimental and dimensional theoretical displacements for the internal resonance and non-resonant systems, respectively. It can be observed that in the non-resonant system, the discrepancy between theory and experiment is less than 10%. In contrast, the internal resonance system shows larger errors, primarily due to the difficulty of achieving perfect internal resonance conditions. Nonetheless, the experiments are conducted to match theoretical assumptions as closely as possible. The primary aim of this study is to evaluate the effect of slapping on the system’s voltage generation.

#### 4.2.3. Experimental and Theoretical Validation of the System’s Voltage Output

**Figure 35:**
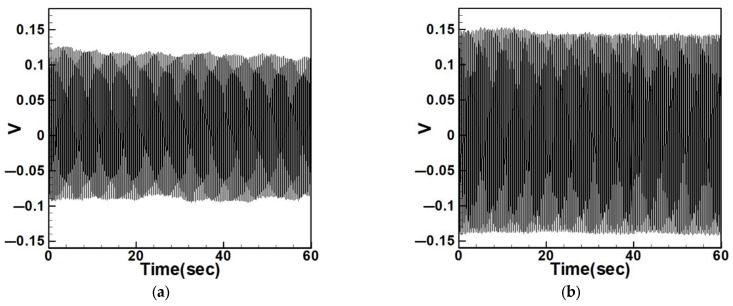
Output voltage of the internal resonance system under first-mode excitation, (**a**) without slapping, (**b**) with slapping.

**Figure 36:**
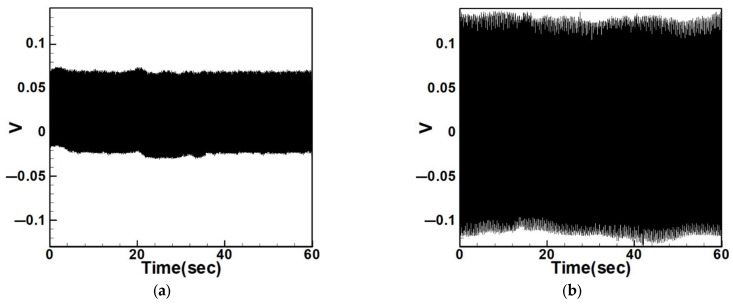
Output voltage of the internal resonance system under third-mode excitation, (**a**) without slapping, (**b**) with slapping.

After verifying the system’s natural frequencies and displacements, this section aims to validate whether the theoretical voltage aligns with the actual output voltage and to compare the effects of slapping force on voltage output. Figure 35 and Figure 36 show the output voltages of the system with internal resonance. It can be observed that the voltage significantly increases and becomes more stable after applying slapping forces. The root-mean-square (RMS) values of these data are compared with theoretical values and summarized in Table 10 and Table 11, with calculated errors.

**Figure 37:**
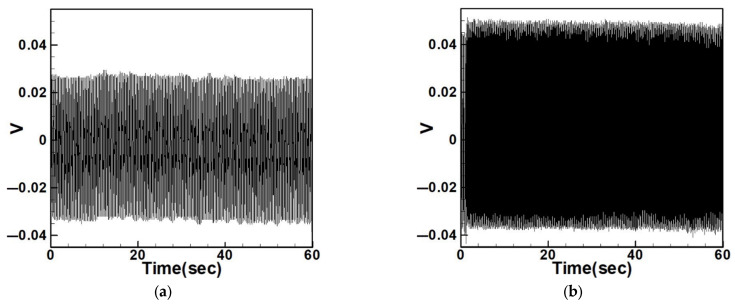
Output voltage of the 5 cm diameter plate without internal resonance under excitation of the first mode, (**a**) without slapping, (**b**) with slapping.

**Figure 38:**
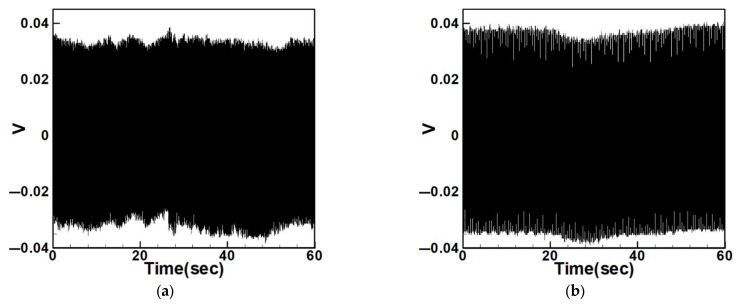
Output voltage of the 5 cm diameter plate without internal resonance under excitation of the second mode, (**a**) without slapping, (**b**) with slapping.

**Figure 39:**
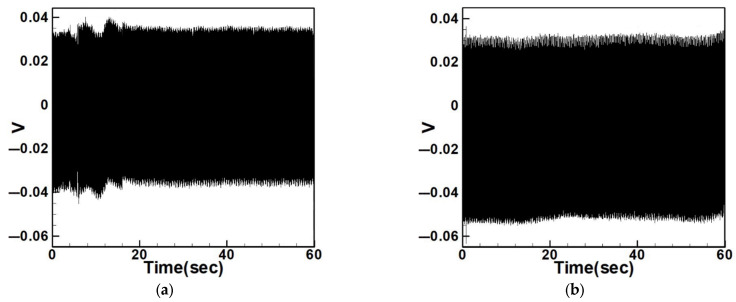
Output voltage of the 5 cm diameter plate without internal resonance under excitation of the third mode, (**a**) without slapping, (**b**) with slapping.

**Figure 40:**
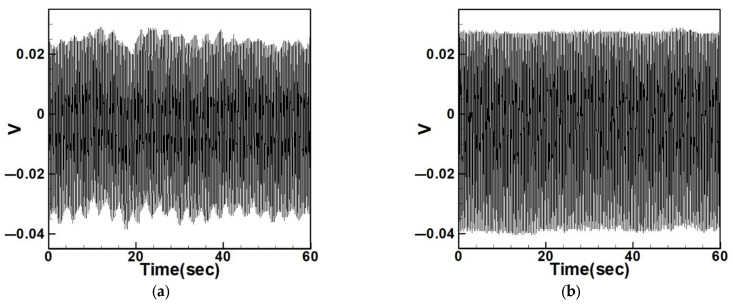
Output voltage of the 10 cm diameter plate without internal resonance under excitation of the first mode, (**a**) without slapping, (**b**) with slapping.

**Figure 41:**
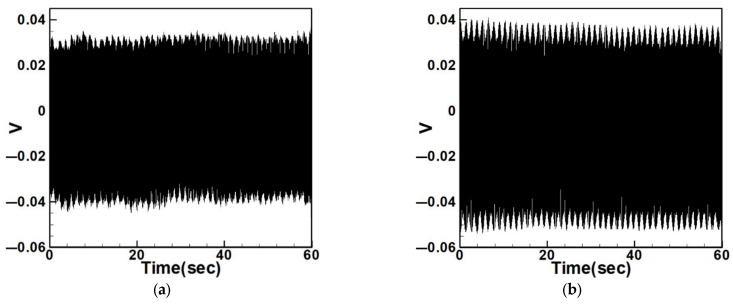
Output voltage of the 10 cm diameter plate without internal resonance under excitation of the second mode, (**a**) without slapping, (**b**) with slapping.

**Figure 42:**
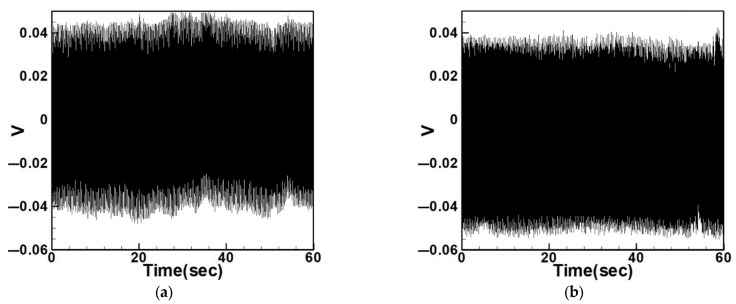
Output voltage of the 10 cm diameter plate without internal resonance under excitation of the third mode, (**a**) without slapping, (**b**) with slapping.

To compare with the impact of internal resonance on voltage generation, Figure 37, Figure 38, Figure 39, Figure 40, Figure 41 and Figure 42 show the experimental output voltages for the first three modes in systems without internal resonance using 5 cm and 10 cm diameter circular plates.

Table 12 and Table 13 list the output voltages for both non-slapping and slapping conditions under the first three modes of excitation in systems without internal resonance. The data show a clear increase in voltage when slapping force is applied, and the errors between the theoretical and experimental results are within 5%.

## 5. Discussion and Analysis of Experimental Results

This section compares the theoretical and experimental results for systems with and without internal resonance by examining natural frequencies, displacement responses, and output voltages. Although some discrepancies exist, most deviations are within 10%, confirming the theoretical model’s predictive accuracy. To further clarify the system’s physical behavior, a deeper quantitative and qualitative discussion is provided below.

### 5.1. Quantitative Discussion and Comparison with Experimental Results

To provide a clearer quantitative understanding of the BIS-VEHS performance, experimental results were analyzed under both internal resonance and non-resonant conditions. Table 14 and Table 15 summarize the RMS voltages and corresponding relative power gains for cases with and without the slapping mechanism.

(1) Internal Resonance Case

Under internal resonance (Table 10 and Table 11), the experimental RMS voltages are summarized in Table 14. The inclusion of the slapping mechanism significantly amplifies the electrical response. For first-mode excitation, the RMS voltage increases from 0.0747 V to 0.1041 V, representing a 39% increase in voltage and nearly 1.94× higher average power for the same load. For third-mode excitation, the improvement is even more pronounced—from 0.0503 V to 0.0866 V—corresponding to a 72% increase in voltage and almost 3× power output. These results demonstrate the synergistic enhancement arising from the coupling between internal resonance and impact-induced slapping. Energy transfer between the first and third modes intensifies strain in the piezoelectric patch, yielding substantially improved harvesting efficiency.

(2) Non-Resonant Case

In contrast, the non-resonant experiments (Table 12 and Table 13) exhibit smaller improvements, as summarized in Table 15. The slapping mechanism still enhances the RMS voltage by 8–23%, leading to 17–50% higher relative power output depending on plate diameter and vibration mode. For example, for a 5 cm plate in first-mode excitation, the voltage rises from 0.0194 V to 0.0238 V, corresponding to a 50% increase in power. Although the slapping effect remains beneficial, the overall enhancement is less dramatic than in the internal resonance case, confirming that mode coupling is the dominant contributor to energy amplification.

To benchmark the BIS-VEHS against other recent nonlinear and hybrid vibration energy harvesters, Table 16 summarizes the RMS voltage levels and relative power ratios obtained in this study alongside representative results from the literature. Under internal resonance with slapping, the BIS-VEHS achieves an RMS voltage of 0.104 V, which is about 1.94× higher power than the configuration without slapping. This relative enhancement is comparable to or greater than those observed in other bistable or tristable VEH systems operating in similar low-frequency ranges. The results confirm that the synergistic combination of internal resonance coupling and slapping impact provides a competitive and bio-inspired route to improved low-frequency energy harvesting.

Table 16 provides a comparative overview of the proposed BIS-VEHS and several representative vibration energy harvesters recently reported in the literature. The BIS-VEHS exhibits a distinctive advantage in low-frequency excitation (5–20 Hz), producing an RMS voltage of 0.104 V, which corresponds to nearly 1.9× higher power than the configuration without slapping. In contrast, the liquid-coupled harvester by Li et al. [28] efficiently captures ultra-low-frequency vibrations below 3 Hz but suffers from limited structural robustness and small effective strain areas. Zhang et al. [29] developed a magnet-free quad-stable piezoelectric harvester based on geometric nonlinearity and bifurcation theory, achieving broadband low-frequency operation (approximately 5–17 Hz) with an output power near 1 mW. This design effectively broadens the frequency bandwidth without relying on magnetic coupling. In contrast, the proposed BIS-VEHS advances this concept by combining internal resonance coupling with a slapping mechanism, enabling simpler geometry, fewer potential wells, and enhanced energy conversion efficiency under similar low-frequency excitation. Consequently, the BIS-VEHS offers a more compact, robust, and application-friendly solution for self-powered sensing applications. Similarly, the tristable electromagnetic design of Chen et al. [19] offers high current output and wide bandwidth but requires a heavier configuration and precise magnetic alignment. Compared with these systems, the BIS-VEHS achieves competitive voltage and relative power levels using a single lightweight piezoelectric element and a simple slapping-coupled structure. The combination of internal resonance energy transfer and impact-induced strain amplification enables strong performance enhancement without magnetic or fluidic components. This synergy allows the BIS-VEHS to maintain high conversion efficiency under low-frequency, low-amplitude excitations that are typical of ambient mechanical environments. Consequently, the proposed device offers a compact and bio-inspired alternative to existing bistable and hybrid harvesters, with clear potential for self-powered sensing and IoT applications.

### 5.2. Physical Discussion and Mechanism Interpretation

Comparison between Table 14 and Table 15 clearly indicates that internal resonance combined with slapping yields two to three-fold higher power output, whereas slapping alone under non-resonant excitation provides only moderate gains. This quantitative evidence confirms that the BIS-VEHS achieves superior energy-conversion efficiency when its structural parameters are tuned to satisfy the internal resonance condition. The experimental results also align closely with theoretical predictions presented in Section 4, validating the proposed model for low-frequency, high-efficiency energy harvesting.

(1) Energy transfer and internal resonance effects.

The frequency response of the internal resonance system (Figure 24a) shows pronounced peaks at low frequencies and suppressed high-frequency modes, consistent with the energy transfer mechanism described by Nayfeh and Mook [14]. The observed multi-peak response also agrees with the nonlinear dynamic behaviors reported by Zhang et al. [22], confirming that internal resonance enhances the energy-harvesting potential of the structure.

(2) Voltage enhancement through slapping mechanism.

From Table 10, Table 11, Table 12 and Table 13, the addition of the slapping mechanism clearly increases voltage output under identical excitation conditions. The slapping force intensifies vibration and simultaneously improves the strain energy conversion efficiency of the piezoelectric patch. Even in non-resonant systems, the slapping-enhanced design outperforms conventional harvesters that neglect impact effects.

(3) Error sources and system sensitivity.

Most theoretical–experimental deviations remain under 10%. Slightly larger discrepancies in the internal resonance cases may arise from material nonlinearity, boundary condition deviations, or variations in slapping force. Because the system is highly sensitive to coupling between slapping force and modal behavior, improved control of impact parameters could further reduce these errors.

(4) Application potential and optimization.

Integrating internal resonance structures with a slapping mechanism not only boosts energy-harvesting efficiency but also broadens the applicability of VEH systems in low-frequency and random vibration environments. Further optimization of slapping frequency, modal coupling, and material properties could enable compact, self-sustaining harvesters for practical deployment.

(5) Mechanism of Energy Transfer and Slapping Interaction.

The internal resonance condition (approximately 1:3 frequency ratio between the first and third modes) enables nonlinear modal energy exchange, where vibration energy initially stored in a higher-order mode is periodically transferred to a lower mode with greater deformation amplitude. This process effectively amplifies strain in the region bonded with the piezoelectric patch, resulting in higher charge generation efficiency. The slapping mechanism further enhances this process by introducing intermittent impact forces that momentarily increase system stiffness and accelerate energy redistribution among modes. Each slapping event excites transient high-frequency components that interact with the internal resonance coupling, thereby sustaining large-amplitude oscillations at low frequencies. The synergy between modal energy transfer and impact-induced strain amplification explains the significant increase in RMS voltage and power output observed experimentally under the resonant condition.

### 5.3. Sensitivity Discussion of Key Parameters

To clarify how structural and operational parameters influence BIS-VEHS performance, a qualitative sensitivity analysis was conducted based on theoretical modeling and experimental trends. Three major parameters—beam stiffness, plate diameter, and slapping period—play central roles in the system’s dynamic response. (1) Beam stiffness (*EI*): Increasing the beam’s bending stiffness raises the system’s natural frequencies and can weaken the internal resonance coupling between modes, thereby reducing vibration amplitude and harvested voltage. Conversely, a more flexible beam promotes stronger modal interaction and strain transfer but may introduce instability or excessive deformation. Hence, a moderate stiffness offers the best balance between mechanical robustness and effective energy conversion. (2) Plate diameter (*D*): Experimental results (Table 12 and Table 13) show that enlarging the circular plate lowers the modal frequency yet reduces the vibration amplitude and output voltage. Smaller plates yield stronger deflection under the same excitation, while larger ones favor frequency matching but with reduced strain. These observations suggest that small-sized plates are optimal for compact, low-frequency harvesters. (3) Frequency sensitivity under internal and non-internal resonance: The frequency response of the BIS-VEHS is strongly governed by whether internal resonance conditions are satisfied. In the non-resonant state, each vibration mode operates independently, and the electrical output peaks sharply near its natural frequency. A small detuning of the excitation frequency causes a rapid drop in displacement amplitude and voltage, indicating a narrow operational bandwidth. In contrast, under internal resonance, the nonlinear coupling between the first and third modes enables energy transfer from higher-mode motion into the lower-frequency mode where the piezoelectric patch is most effective. This inter-modal energy exchange allows the system to maintain a relatively stable voltage output even when the excitation frequency deviates slightly from the resonance point, effectively broadening the harvesting bandwidth. Consequently, internal resonance not only enhances energy conversion efficiency but also improves frequency robustness, making the BIS-VEHS more adaptable to variable vibration environments.

### 5.4. Future Perspectives

In future work, several extensions of the present BIS-VEHS study can be pursued.

(1) Structural optimization—The geometry and material parameters of the circular plate and elastic beam can be further optimized using topology or machine-learning-based design to maximize power density under low-frequency excitation.

(2) Integration with sensor networks—The proposed harvester can be coupled with ultra-low-power wireless sensors to form a self-powered sensing node for structural-health monitoring or environmental data collection.

(3) Multiphysics modeling—Future analyses should incorporate fluid–structure interaction and nonlinear damping to more accurately represent real environmental excitations such as wind or flow-induced vibration.

(4) Power-management circuitry—Experimental studies on rectification, energy storage, and load matching will help quantify the overall conversion efficiency of the BIS-VEHS in practical systems.

(5) Bio-inspired scaling and hybrid mechanisms—Expanding the slapping concept to arrays or combining it with magneto-electric or triboelectric modules could yield broadband, multi-mode energy harvesting suitable for next-generation IoT devices. These investigations will advance the BIS-VEHS concept from laboratory demonstration to real-world, self-sustaining sensor applications.

## 6. Conclusions

This study proposed and experimentally validated an epiphytic plant bio-inspired slapping vibration energy harvesting system (BIS-VEHS) that integrates internal resonance and impact-induced slapping to enhance low-frequency energy conversion efficiency. Both theoretical modeling and experimental measurements confirm the strong coupling between the system’s nonlinear dynamics and its power generation capability.

The main conclusions are summarized as follows:

1. Validation of theoretical modeling.

The nonlinear dynamic model accurately predicts the system behavior, with theoretical–experimental discrepancies generally within 10% for displacement and voltage responses. This confirms that the simplified mathematical formulation captures the essential physics of the BIS-VEHS.

2. Influence of internal resonance.

Experiments demonstrate that the 1:3 internal resonance coupling effectively transfers energy from higher-order to lower-order modes. Under this condition, the harvested power increases by nearly two- to three-fold compared with the non-resonant case, verifying that modal energy transfer is a key mechanism for improved efficiency.

3. Role of the slapping mechanism.

The inclusion of the slapping element significantly amplifies strain in the piezoelectric layer. Even without internal resonance, slapping produces 17–50% higher power output; when combined with internal resonance, it yields nearly three times the harvested power. These results confirm the synergistic function of mechanical impact and nonlinear modal interaction.

4. Experimental verification and robustness.

The measured frequency responses exhibit multiple peaks and amplitude modulation consistent with nonlinear dynamic theory. The BIS-VEHS maintains stable performance across different plate diameters and excitation modes, demonstrating reliable operation under low-frequency and random vibration conditions.

5. Practical significance and applications.

The combination of internal resonance structures with slapping mechanisms provides a compact and efficient platform for self-powered sensors and IoT devices operating in low-frequency environments such as pipelines, bridges, or machinery housings. The design concept can be further optimized through geometry tuning, smart-material selection, and integrated power-management circuitry.

In summary, the proposed BIS-VEHS represents a novel hybrid energy-harvesting architecture that merges biomimetic inspiration with nonlinear vibration dynamics. Its experimentally validated improvements in voltage and power output demonstrate strong potential for real-world applications where compact, broadband, and self-sustaining power sources are required.

## Figures and Tables

**Table 1:** Internal resonance conditions for three different circular plate diameters.

Circular Plate Dimensionless Diameters	${\bar{I}}_{b}$	$\omega_{1}$	$\omega_{3}$
0.4	0.19294	0.15584	0.46751
0.8	0.12930	0.13630	0.40891
1.2	0.12431	0.12595	0.37784

**Table 2:** Maximum amplitudes with internal resonance.

	D = 0.4		D = 0.8		D = 1.2	
	Excite 1st Mode	Excite 3rd Mode	Excite 1st Mode	Excite 3rd Mode	Excite 1st Mode	Excite 3rd Mode
Mode 1	0.27612	0.24564	0.1966	0.1838	0.188	0.1232
Mode 3	0.06524	0.1368	0.04972	0.0978	0.0474	0.06828

**Table 3:** Maximum amplitudes in the non-internal resonant system.

	D = 0.4	D = 0.8	D = 1.2
Excite 1st mode	0.11912	0.11832	0.10664
Excite 2nd mode	0.06736	0.05888	0.05808
Excite 3rd mode	0.04016	0.03224	0.03056

**Table 4:** The 1st mode output voltages with and without slapping force under internal resonance.

Diameter Ratio	D = 0.4		D = 0.8		D = 1.2	
Mode excited	1st	3rd	1st	3rd	1st	3rd
*V_No Slp_*	0.00694	0.00475	0.00361	0.00292	0.00313	0.00240
*V_slp_*	0.00872	0.00674	0.00489	0.00451	0.00460	0.00346

**Table 5:** Output voltages (dimensionless) with and without slapping force under non-resonant conditions.

	Diameter Ratio	D = 0.4	D = 0.8	D = 1.2
*V_No Slp_*	Excite 1st mode	0.00160	0.00148	0.00122
	Excite 2nd mode	0.00183	0.00171	0.00141
	Excite 3rd mode	0.00193	0.00186	0.00152
*V_slp_*	Excite 1st mode	0.00192	0.00177	0.00146
	Excite 2nd mode	0.00203	0.00199	0.00161
	Excite 3rd mode	0.00219	0.00209	0.00180

**Table 6:** Comparison of dimensional theoretical and experimental natural frequencies for the internal resonance system.

	Excite 1st	Excite 3rd
Theo.	4.36	13.08
Exp.	4.25	12.45
Error (%)	2.52	4.82

**Table 7:** Comparison of dimensional theoretical and experimental natural frequencies for the non-resonant system.

	D = 5 cm			D = 10 cm		
	Excite 1st	Excite 2nd	Excite 3rd	Excite 1st	Excite 2nd	Excite 3rd
Theo.	4.41	8.51	13.56	4.02	9.87	15.83
Exp.	4.15	8.19	13.15	4.07	9.57	15.65
Error (%)	5.90	3.76	3.02	1.24	3.04	1.14

**Table 8:** Comparison of RMS values (mm) between theoretical and experimental displacements for the internal resonance system (dimensional data).

	Excite 1st Mode	Excite 3rd Mode
Theo.	19.684126	20.27122
Exp.	22.351167	22.420755
Error (%)	13.549196	10.603875

**Table 9:** Comparison of RMS values (mm) between theoretical and experimental displacements for the non-resonant system (dimensional data).

	D = 5 cm			D = 10 cm		
	Excite 1st Mode	Excite 2nd Mode	Excite 3rd Mode	Excite 1st Mode	Excite 2nd Mode	Excite 3rd Mode
Theo.	10.5639	5.9358	3.5290	10.5425	5.2164	2.7329
Exp.	9.9936	5.8203	3.3733	10.1664	4.9504	2.6161
Error (%)	5.3987	1.9450	4.4114	3.5676	5.0997	4.2755

**Table 10:** Comparison of theoretical and experimental RMS voltage without slapping (1st mode, internal resonance).

	Excite 1st Mode	Excite 3rd Mode
Theo. (V)	0.086796	0.059363
Exp. (V)	0.074732	0.050280
Error (%)	13.898451	15.299765

**Table 11:** Comparison of theoretical and experimental RMS voltage with slapping (1st mode, internal resonance).

	Excite 1st Mode	Excite 3rd Mode
Theo. (V)	0.108962	0.084209
Exp. (V)	0.104095	0.0865736
Error (%)	4.466694811	2.808013395

**Table 12:** Comparison of theoretical and experimental RMS voltage without slapping (1st mode, no internal resonance).

	D = 5 cm			D = 10 cm		
	Excite 1st Mode	Excite 2nd Mode	Excite 3rd Mode	Excite 1st Mode	Excite 2nd Mode	Excite 3rd Mode
Theo. (V)	0.01999	0.02291	0.02408	0.01846	0.02142	0.02330
Exp. (V)	0.01944	0.02317	0.02527	0.01847	0.02110	0.02406
Error (%)	2.71795	1.14112	4.92878	0.00649	1.49383	3.26509

**Table 13:** Comparison of theoretical and experimental RMS voltage with slapping (1st mode, no internal resonance).

	D = 5 cm			D = 10 cm		
	Excite 1st Mode	Excite 2nd Mode	Excite 3rd Mode	Excite 1st Mode	Excite 2nd Mode	Excite 3rd Mode
Theo. (V)	0.02394	0.02533	0.02742	0.02218	0.02490	0.02610
Exp. (V)	0.02383	0.02506	0.02872	0.02136	0.02500	0.02626
Error (%)	0.48824	1.06535	4.75635	3.68407	0.40882	0.62182

**Table 14:** Experimental RMS voltage and relative power gain under internal resonance.

Excited Mode	V_no slapping_ (V)	V_with slapping_ (V)	ΔV (%)	P_slap_/P_no_	ΔP (%)
1st mode	0.07473	0.10409	39.3	1.94	94.0
3rd mode	0.05028	0.08657	72.2	2.96	196.5

**Table 15:** Experimental RMS voltage and relative power gain without internal resonance.

Plate Dia.	Excited Mode	$V_{\mathrm{no}\;\mathrm{slapping}}$(V)	$V_{\mathrm{with}\;\mathrm{slapping}}$(V)	ΔV (%)	$P_{\mathrm{slap}}/P_{\mathrm{no}}$	ΔP (%)
5 cm	1st	0.01944	0.02383	22.6	1.50	50.3
5 cm	2nd	0.02317	0.02506	8.2	1.17	17.0
5 cm	3rd	0.02527	0.02872	13.7	1.29	29.2
10 cm	1st	0.01847	0.02136	15.6	1.34	33.7
10 cm	2nd	0.02110	0.02500	18.5	1.40	40.4
10 cm	3rd	0.02406	0.02626	9.1	1.19	19.1

**Table 16:** Comparison of the proposed BIS-VEHS with representative vibration energy harvesters (2023–2024).

System/Reference	Frequency Range (Hz)	RMS Voltage/Power Trend	Key Mechanism	Remarks
Proposed BIS-VEHS (with slapping)	5~20	0.104 V (RMS), ≈1.9× higher power than no-slap case	Internal resonance + slapping impact	High efficiency at low frequency; bio-inspired design
Piezoelectric (liquid-coupled cantilever), Li et al., *Micromachines* 2023, [28]	<3	≈0.07 V RMS (~51 µW at 0.03 g)	Liquid-medium energy capture	Ultra-low-frequency multidirectional harvesting
Quad-stable PEH, Zhang et al., *Sensors* 2022, [29]	~5–17 Hz	~0.1 V RMS (≈1 mW peak)	Geometric nonlinear piezoelectric harvester (bifurcation-based)	Broadband low-frequency, geometric nonlinearity, magnet-free design
Electromagnetic, tristable, Chen et al., *Energy Reports* 2024, [19]	20~30	0.1 V RMS	Dual helical spring stiffness + Halbach arrays	Broadband, small-amplitude low-frequency operation

## Data Availability

The data presented in this study are available upon reasonable request from the corresponding author.

## Nomenclature

Symbol	Description
*A*	Circle plate (leaf) cross-sectional area
*A_b_*	beam cross-sectional area
*C_p_*	Capacitance of the PZT
*C_f_*	Piezoelectric coupling coefficient
E	Young’s modulus of the elastic beam
*F*	Slapping force (acting on the root of the main structure)
*F_b_*	Wind force acting on both the circular plate and the rectangular plate.
I	Beam area moment of inertia
${\bar{I}}_{A}$	Dimensionless mass moment of inertia of the circle plate (leaf)
${\bar{I}}_{b}$	$\frac{m_{b}y^{2}}{m_{b}l^{2}}$ Dimensionless beam mass moment of inertia
l	Length of beam
$\bar{M}$	Mass ratio of the circle plate to the beam
*m_b_*	beam mass
*q*	distributed external force
$\bar{q}$	Dimensionless distributed external force
*T*	Slapping period
$\bar{T}$	Dimensionless slapping period
*t*	Dimensional time
*V*	Voltage
$\alpha_{n}$	Eigenvalue of *n*^th^ mode
ε	Time scale of small perturbations.
η	Von Kármán nonlinear strain
$\mu_{w}$	Dimensionless damping coefficient
ξ	Generalized coordinate (time-dependent variable)
ν	Dimensionless voltage
ρ	Density
τ	Dimensionless time
$\phi_{n}$	Mode shape of *n*^th^ mode
σ	Tuned frequency
$\zeta_{n}$	The *n*^th^ mode phase
$\omega_{n}$	Natural frequency of *n*^th^ mode

## Appendix A

To understand the vibration behavior of this system, the vibration modes are analyzed. First, using the method of separation of variables, the transverse deformation ${\bar{w}}_{0}$ is defined as follows:

(A1) $${\bar{w}}_{0}=X\left(\bar{x}\right)Y\left(\tau \right)$$

Assuming the general solution for $X\left(\bar{x}\right)$ is

(A2) $$X(\bar{x})=E_{1}\cos\alpha \bar{x}+E_{2}\cosh\alpha \bar{x}+E_{3}\sin\alpha \bar{x}+E_{4}\sinh\alpha \bar{x}$$

The boundary conditions for $\bar{w}$ are expressed as follows: $\bar{w}\left(0,\tau \right)=0$, $\bar{w^{\prime }}\left(0,\tau \right)=0$, $\bar{w^{\prime \prime }}\left(1,\tau \right)=\alpha^{4}{\bar{I}}_{A}\bar{w^{\prime }}$, ${\bar{w}}^{\prime \prime \prime }\left(1,\tau \right)=-\alpha^{4}\bar{M}X$, where α is the eigenvalue, ${\bar{I}}_{A}$ is the dimensionless mass moment of inertia of the circle plate (leaf), and $\bar{M}$ is the mass ratio of the circle plate to the beam. Substituting the boundary conditions into Equation (A2) and performing calculations and processing yields the following characteristic equation:

(A3) $$\left[\alpha^{2}\sin\alpha +\alpha^{2}\sinh\alpha +{\bar{I}}_{A}\alpha^{5}\cos\alpha -{\bar{I}}_{A}\alpha^{5}\cosh\alpha \right]\;\left[\alpha^{3}\sin\alpha -\alpha^{3}\sinh\alpha +\bar{M}\alpha^{4}\cos\alpha -\bar{M}\alpha^{4}\cosh\alpha \right]\;\;+\left[\alpha^{2}\cos\alpha +\alpha^{2}\cosh\alpha -{\bar{I}}_{A}\alpha^{5}\sin\alpha -{\bar{I}}_{A}\alpha^{5}\sinh\alpha \right]\;\left[\alpha^{3}\cos\alpha +\alpha^{3}\cosh\alpha -\bar{M}\alpha^{4}\sin\alpha +\bar{M}\alpha^{4}\sinh\alpha \right]=0$$

The vibration mode shapes of this system can then be obtained as follows:

(A4) $$\phi_{n}=\left[\cos(\alpha_{n}\bar{x})-\cosh(\alpha_{n}\bar{x})\right]\;+\frac{-\cos\alpha_{n}-\cosh\alpha_{n}+{\bar{I}}_{A}\alpha_{n}^{3}\sin\alpha_{n}+{\bar{I}}_{A}\alpha_{n}^{3}\sinh\alpha_{n}}{\sin\alpha_{n}+\sinh\alpha_{n}+{\bar{I}}_{A}\alpha_{n}^{3}\cos\alpha_{n}-{\bar{I}}_{A}\alpha_{n}^{3}\cosh\alpha_{n}}\left[\sin\alpha_{n}\bar{x}\right.\;\left.-\sinh\alpha_{n}\bar{x}\right]$$

## Appendix B

First, consider the case when the first mode is excited ($q_{1}e^{i\Omega \tau }=q_{1}e^{i\sigma T_{1}}e^{i\omega_{1}T_{0}}$). Multiply Equation (26) by $e^{i\zeta_{1}}$, and let $\Gamma_{A}=\sigma T_{1}+\zeta_{1}$ and $\Gamma_{B}=3\zeta_{1}-\zeta_{3}$. Then, separate the equation into its real and imaginary components to satisfy the solvability conditions.

The real part of the first mode is given by

(A5) $$\begin{array}{l} -2\omega_{1}\zeta_{1}^{\prime }B_{1}+2{\bar{I}}_{b}\omega_{1}\zeta_{1}^{\prime }B_{1}\frac{\int_{0}^{1}\phi_{1}^{\prime \prime }\phi_{1}d\bar{x}}{\int_{0}^{1}\phi_{1}^{2}d\bar{x}}+\omega_{1}^{2}\left[\frac{3}{2}B_{1}^{2}{\bar{B}}_{1}\frac{\int_{0}^{1}\phi_{1}^{\prime \prime }\phi_{1}^{\prime 2}\phi_{1}d\bar{x}}{\int_{0}^{1}\phi_{1}^{2}d\bar{x}}+{\bar{B}}_{1}^{2}B_{3}\cos\left(\Gamma_{B}\right)\frac{\int_{0}^{1}\phi_{1}^{\prime \prime }\phi_{1}^{\prime }\phi_{3}^{\prime }\phi_{1}d\bar{x}}{\int_{0}^{1}\phi_{1}^{2}d\bar{x}}\right.\\ +B_{1}B_{3}{\bar{B}}_{3}\frac{\int_{0}^{1}\phi_{1}^{\prime \prime }\phi_{3}^{\prime 2}\phi_{1}d\bar{x}}{\int_{0}^{1}\phi_{1}^{2}d\bar{x}}\\ +\frac{1}{2}{\bar{B}}_{1}^{2}B_{3}\cos\left(\Gamma_{B}\right)\frac{\int_{0}^{1}\phi_{3}^{\prime \prime }\phi_{1}^{\prime 2}\phi_{1}d\bar{x}}{\int_{0}^{1}\phi_{3}\phi_{1}d\bar{x}}\;\left.+2B_{1}B_{3}{\bar{B}}_{3}\frac{\int_{0}^{1}\phi_{3}^{\prime \prime }\phi_{1}^{\prime }\phi_{3}^{\prime }\phi_{1}d\bar{x}}{\int_{0}^{1}\phi_{3}\phi_{1}d\bar{x}}\right]=-{\bar{q}}_{1}\cos\left(\Gamma_{A}\right)\frac{\int_{0}^{1}\phi_{1}d\bar{x}}{\int_{0}^{1}\phi_{1}^{2}d\bar{x}} \end{array}$$

The imaginary part of the first mode is given by

(A6) $$-2\omega_{1}B_{1}^{\prime }+2{\bar{I}}_{b}\omega_{1}B_{1}^{\prime }\frac{\int_{0}^{1}\phi_{1}^{\prime \prime }\phi_{1}d\bar{x}}{\int_{0}^{1}\phi_{1}^{2}d\bar{x}}+\omega_{1}^{2}\left[{\bar{B}}_{1}^{2}B_{3}\sin\left(\Gamma_{B}\right)\frac{\int_{0}^{1}\phi_{1}^{\prime \prime }\phi_{1}^{\prime }\phi_{3}^{\prime }\phi_{1}d\bar{x}}{\int_{0}^{1}\phi_{1}^{2}d\bar{x}}\right.\;\;\left.+\frac{1}{2}{\bar{B}}_{1}^{2}B_{3}\sin\left(\Gamma_{B}\right)\frac{\int_{0}^{1}\phi_{3}^{\prime \prime }\phi_{1}^{\prime 2}\phi_{1}d\bar{x}}{\int_{0}^{1}\phi_{3}\phi_{1}d\bar{x}}\right]-\mu_{w}\omega_{1}B_{1}=-{\bar{q}}_{1}\sin\left(\Gamma_{A}\right)\frac{\int_{0}^{1}\phi_{1}d\bar{x}}{\int_{0}^{1}\phi_{1}^{2}d\bar{x}}$$

Next, when the third mode is excited. Multiply Equation (A5) by $e^{i\zeta_{3}}$ and follow the same procedure as before.

The real part of the third mode is given by

(A7) $$\begin{array}{l} -2\omega_{3}\zeta_{3}^{\prime }B_{3}+2{\bar{I}}_{b}\omega_{3}\zeta_{3}^{\prime }B_{3}\frac{\int_{0}^{1}\phi_{3}^{\prime \prime }\phi_{3}d\bar{x}}{\int_{0}^{1}\phi_{3}^{2}d\bar{x}}+\omega_{3}^{2}\left[\frac{1}{2}B_{1}^{3}\cos\left(-\Gamma_{B}\right)\frac{\int_{0}^{1}\phi_{1}^{\prime \prime }\phi_{1}^{\prime }\phi_{1}^{\prime }\phi_{3}d\bar{x}}{\int_{0}^{1}\phi_{1}\phi_{3}d\bar{x}}\right.+2B_{1}{\bar{B}}_{1}B_{3}\frac{\int_{0}^{1}\phi_{1}^{\prime \prime }\phi_{1}^{\prime }\phi_{3}^{\prime }\phi_{3}d\bar{x}}{\int_{0}^{1}\phi_{1}\phi_{3}d\bar{x}}\\ +B_{1}{\bar{B}}_{1}B_{3}\frac{\int_{0}^{1}\phi_{3}^{\prime \prime }\phi_{1}^{\prime }\phi_{1}^{\prime }\phi_{3}d\bar{x}}{\int_{0}^{1}\phi_{3}^{2}d\bar{x}}\;\left.+\frac{3}{2}B_{3}^{2}{\bar{B}}_{3}\frac{\int_{0}^{1}\phi_{3}^{\prime \prime }\phi_{3}^{\prime }\phi_{3}^{\prime }\phi_{3}d\bar{x}}{\int_{0}^{1}\phi_{3}\phi_{3}d\bar{x}}\right]=0 \end{array}$$

The imaginary part of the third mode is given by

(A8) $$-2\omega_{3}B_{3}^{\prime }+2{\bar{I}}_{b}\omega_{3}B_{3}^{\prime }\frac{\int_{0}^{1}\phi_{3}^{\prime \prime }\phi_{3}d\bar{x}}{\int_{0}^{1}\phi_{3}^{2}d\bar{x}}+\frac{\omega_{3}^{2}}{2}B_{1}^{3}\sin\left(-\Gamma_{B}\right)\frac{\int_{0}^{1}\phi_{1}^{\prime \prime }\phi_{1}^{\prime }\phi_{1}^{\prime }\phi_{3}d\bar{x}}{\int_{0}^{1}\phi_{1}\phi_{3}d\bar{x}}-\mu_{w}\omega_{3}B_{3}=0$$

In the next step, this study will demonstrate the procedure for obtaining the fixed-point plot when the first mode is excited. Since *B_n_* represents the amplitude of the *n*^th^ mode of the system, and *B_n_* is a constant, its derivative equals zero. To determine the frequency response of the system, let $\Gamma_{A}^{\prime }=\sigma +\zeta_{1}^{\prime }=0\Rightarrow \zeta_{1}^{\prime }=-\sigma$ and $\Gamma_{B}^{\prime }=3\zeta_{1}^{\prime }-\zeta_{3}^{\prime }=0$ $\Rightarrow 3\zeta_{1}^{\prime }=\zeta_{3}^{\prime }=-3\sigma$ and substitute these into the real and imaginary parts of the first and third modes. To eliminate the time-dependent terms, square and sum the real and imaginary parts of the first mode, which is given by

(A9) $$\left[2\omega_{1}\sigma B_{1}-2{\bar{I}}_{b}\omega_{1}\sigma B_{1}\frac{\int_{0}^{1}\phi_{1}^{\prime \prime }\phi_{1}d\bar{x}}{\int_{0}^{1}\phi_{1}^{2}d\bar{x}}+\omega_{1}^{2}\left(\frac{3}{2}B_{1}^{2}{\bar{B}}_{1}\frac{\int_{0}^{1}\phi_{1}^{\prime \prime }\phi_{1}^{\prime 2}\phi_{1}d\bar{x}}{\int_{0}^{1}\phi_{1}^{2}d\bar{x}}\right.\right.\;+{\bar{B}}_{1}^{2}B_{3}\cos\left(\Gamma_{B}\right)\frac{\int_{0}^{1}\phi_{1}^{\prime \prime }\phi_{1}^{\prime }\phi_{3}^{\prime }\phi_{1}d\bar{x}}{\int_{0}^{1}\phi_{1}^{2}d\bar{x}}+B_{1}B_{3}{\bar{B}}_{3}\frac{\int_{0}^{1}\phi_{1}^{\prime \prime }\phi_{3}^{\prime 2}\phi_{1}d\bar{x}}{\int_{0}^{1}\phi_{1}^{2}d\bar{x}}\;\;{\left.\left.+\frac{1}{2}{\bar{B}}_{1}^{2}B_{3}\cos\left(\Gamma_{B}\right)\frac{\int_{0}^{1}\phi_{3}^{\prime \prime }\phi_{1}^{\prime 2}\phi_{1}d\bar{x}}{\int_{0}^{1}\phi_{3}\phi_{1}d\bar{x}}+2B_{1}B_{3}{\bar{B}}_{3}\frac{\int_{0}^{1}\phi_{3}^{\prime \prime }\phi_{1}^{\prime }\phi_{3}^{\prime }\phi_{1}d\bar{x}}{\int_{0}^{1}\phi_{3}\phi_{1}d\bar{x}}\right)\right]}^{2}+\left[\omega_{1}^{2}\left({\bar{B}}_{1}^{2}B_{3}\sin\left(\Gamma_{B}\right)\frac{\int_{0}^{1}\phi_{1}^{\prime \prime }\phi_{1}^{\prime }\phi_{3}^{\prime }\phi_{1}d\bar{x}}{\int_{0}^{1}\phi_{1}^{2}d\bar{x}}\right.\right.\;\;{\left.\left.+\frac{1}{2}{\bar{B}}_{1}^{2}B_{3}\sin\left(\Gamma_{B}\right)\frac{\int_{0}^{1}\phi_{3}^{\prime \prime }\phi_{1}^{\prime 2}\phi_{1}d\bar{x}}{\int_{0}^{1}\phi_{3}\phi_{1}d\bar{x}}\right)-\mu_{w}\omega_{1}B_{1}\right]}^{2}={\bar{q}}_{1}^{2}{\left(\frac{\int_{0}^{1}\phi_{1}d\bar{x}}{\int_{0}^{1}\phi_{1}^{2}d\bar{x}}\right)}^{2}$$

The real part of the third mode is given by

(A10) $$\begin{array}{l} 6\omega_{3}\sigma B_{3}-6{\bar{I}}_{b}\omega_{3}\sigma B_{3}\frac{\int_{0}^{1}\phi_{3}^{\prime \prime }\phi_{3}d\bar{x}}{\int_{0}^{1}\phi_{3}^{2}d\bar{x}}+\omega_{3}^{2}\left[\frac{1}{2}B_{1}^{3}\cos\left(-\Gamma_{B}\right)\frac{\int_{0}^{1}\phi_{1}^{\prime \prime }\phi_{1}^{\prime }\phi_{1}^{\prime }\phi_{3}d\bar{x}}{\int_{0}^{1}\phi_{1}\phi_{3}d\bar{x}}\right.\\ +2B_{1}{\bar{B}}_{1}B_{3}\frac{\int_{0}^{1}\phi_{1}^{\prime \prime }\phi_{1}^{\prime }\phi_{3}^{\prime }\phi_{3}d\bar{x}}{\int_{0}^{1}\phi_{1}\phi_{3}d\bar{x}}+B_{1}{\bar{B}}_{1}B_{3}\frac{\int_{0}^{1}\phi_{3}^{\prime \prime }\phi_{1}^{\prime }\phi_{1}^{\prime }\phi_{3}d\bar{x}}{\int_{0}^{1}\phi_{3}^{2}d\bar{x}}\left.+\frac{3}{2}B_{3}^{2}{\bar{B}}_{3}\frac{\int_{0}^{1}\phi_{3}^{\prime \prime }\phi_{3}^{\prime }\phi_{3}^{\prime }\phi_{3}d\bar{x}}{\int_{0}^{1}\phi_{3}\phi_{3}d\bar{x}}\right]=0 \end{array}$$

The imaginary part of the third mode is given by

(A11) $$\frac{\omega_{3}^{2}}{2}B_{1}^{3}\sin\left(-\Gamma_{B}\right)\frac{\int_{0}^{1}\phi_{1}^{\prime \prime }\phi_{1}^{\prime }\phi_{1}^{\prime }\phi_{3}d\bar{x}}{\int_{0}^{1}\phi_{1}\phi_{3}d\bar{x}}-\mu_{w}\omega_{3}B_{3}=0$$

## Appendix C

**Table A1:** Physical and dimensionless parameters used in the simulations and experimental validation of the BIS-VEHS.

Parameter	Symbol	Dimensionless/Normalized Value	Description
Beam length	l	1.0	Reference length for normalization
Circular-plate diameter	D	0.4–0.8	Diameter ratio D/l
Equivalent stiffness	EI	1.0	Baseline stiffness for normalization
Plate mass	$m_{p}$	0.12–0.25	Normalized mass relative to beam mass
Piezoelectric patch length	$l_{p}$	0.16	$L_{p}/L$ ratio
Piezoelectric coupling coefficient	$\hat{k}$	0.002	Dimensionless coupling between mechanical strain and electric field (from PZT-5H properties)
